# The antiviral and immunomodulatory activities of propolis: An update and future perspectives for respiratory diseases

**DOI:** 10.1002/med.21866

**Published:** 2021-11-02

**Authors:** Andrea Magnavacca, Enrico Sangiovanni, Giorgio Racagni, Mario Dell'Agli

**Affiliations:** ^1^ Department of Pharmacological and Biomolecular Sciences University of Milan Milan Italy

**Keywords:** antiviral, inflammation, immunomodulator, propolis, respiratory diseases

## Abstract

Propolis is a complex natural product that possesses antioxidant, anti‐inflammatory, immunomodulatory, antibacterial, and antiviral properties mainly attributed to the high content in flavonoids, phenolic acids, and their derivatives. The chemical composition of propolis is multifarious, as it depends on the botanical sources from which honeybees collect resins and exudates. Nevertheless, despite this variability propolis may have a general pharmacological value, and this review systematically compiles, for the first time, the existing preclinical and clinical evidence of propolis activities as an antiviral and immunomodulatory agent, focusing on the possible application in respiratory diseases. In vitro and in vivo assays have demonstrated propolis broad‐spectrum effects on viral infectivity and replication, as well as the modulatory actions on cytokine production and immune cell activation as part of both innate and adaptive immune responses. Clinical trials confirmed propolis undeniable potential as an effective therapeutic agent; however, the lack of rigorous randomized clinical trials in the context of respiratory diseases is tangible. Since propolis is available as a dietary supplement, possible use for the prevention of respiratory diseases and their deleterious inflammatory drawbacks on the respiratory tract in humans is considered and discussed. This review opens up new perspectives on the clinical investigation of neglected propolis biological properties which, now more than ever, are particularly relevant with respect to the recent outbreaks of pandemic respiratory infections.

## 1 INTRODUCTION

### 1.1 Propolis

Propolis is a resinous complex mixture elaborated by forager honeybees (*Apis mellifera *Linnaeus, 1758) with exudates and resins gathered from different parts of plants (e.g., buds and twigs), mixed with beeswax and salivary enzymes (β‐glucosidase, α/β‐amylase, maltase, esterase, etc.).^1^ The term propolis, from Ancient Greek πϱο‐ (*pro‐*, “in front of/for”) + πόλις (*pólis*, “city”), refers to the important role of this product in the protection of the colony since it is used by honeybees as a building material to line the beehive, seal small openings and cracks, and strengthen the honeycomb. In addition, propolis is used to protect the hive against parasites and predators, preventing infections and microbial growth and impeding the putrefaction of dead intruders that bees are unable to take out.

Propolis is a gluey lipophilic mixture generally composed of about 50% resin, 30% wax, 10% essential oil, 5% pollen, and 5% other substances including a diversity of minerals (calcium, copper, iodine, iron, magnesium, manganese, potassium, sodium, and zinc), vitamins (B1, B2, B6, C, E, D, and pro‐vitamin A), poly‐ and oligo‐saccharides, and phenolic compounds (i.e., flavonoids, aromatic acids, and esters, etc.).^1^ Several propolis samples from different geographical origins have been thoroughly characterized and tentatively classified on the basis of their principal components and putative botanical sources. However, within the same propolis type the chemical composition of specific samples may be highly variable depending on plant ecology, season, climatic factors, and environmental conditions of the site of collection, thus making standardization a great challenge.^2^, ^3^

In Table 1, the most widespread and well‐known propolis types, together with their chemical markers, are presented. Data about propolis collected in Africa, the Middle East, Australia, and to some extent North America are scarce and reveal multifarious chemistry. Hence, the classification of propolis types from these regions is difficult and still incomplete.^4^

**Table 1:** Classification of the principal propolis types (*elaborated from* ^4^, ^5^)

Propolis type	Subtype	Geographical origin	Botanical origin	Components–chemical markers	References
Poplar propolis		Europe, North America, temperate regions of Asia (e.g., China)	*Populus* spp. (sect. *Aigeiros*, most commonly *P. nigra* L.)	Flavonoids, phenolic acids, and their esters – Pinocembrin, chrysin, galangin, pinobanksin, pinobanksin 3‐acetate	^6^, ^7^, ^8^
Aspen propolis		Northern regions of Europe	*Populus tremula* L.	*p*‐coumaric acid, ferulic acid, benzoic acid, benzyl *p*‐coumarate, benzyl ferulate, glycerol esters of substituted cinnamic acids (phenolic glycerides)	^9^, ^10^, ^11^
Birch propolis		Russia	*Betula pendula* Roth	Flavones and flavonols (different from those of poplar propolis)	^12^
Mediterranean propolis		Mediterranean region of Europe	*Cupressus sempervirens* L.	Diterpenes – isocupressic acid, pimaric acid, agathadiol, isoagatholal, totarol. N.B. Usually does not contain flavonoids and phenolic acids	^13^, ^14^
Canarian type propolis		Canary Islands	Unknown	Furofuran lignanes	^15^
Pacific propolis		Taiwan, Okinawa, Hawaii, Indonesia	*Macaranga tanarius* (L.) Müll. Arg.	Prenylated flavanones (propolins)	^16^, ^17^, ^18^
Mangifera indica propolis		Indonesia, Myanmar, Thailand	*Mangifera indica* L.	Phenolic lipids (cardanols, cardols, anacardic acid derivatives)	^18^, ^19^
Caribbean propolis		Cuba, Venezuela	*Clusia* spp. (most commonly *C. rosea* Jacq. and *C. minor* L.)	Polyprenylated benzophenones – Nemorosone, guttiferone E	^20^, ^21^, ^22^
Brazilian propolis	3	Paraná State	*Populus* spp.	Mainly flavonoids – Chrysin, pinocembrin, pinobanksin, apigenin, galangin	^23^, ^24^
	6 Brown	Bahia State	*Hyptis divaricata* Pohl ex Benth.	Prenylated benzophenones	^23^, ^24^, ^25^
	12 Green	São Paulo State	*Baccharis dracunculifolia* DC.	Prenylated phenolic acids and flavonoids – Artepillin C, drupanin, *p*‐coumaric acid, dihydrocinnamic acid	^23^, ^24^, ^26^, ^27^, ^28^
	13 Red	Alagoas State	*Dalbergia ecastaphyllum* (L.) Taub.	Isoflavans, pterocarpans, and chalcones – Vestitol, neovestitol, 7‐O‐methylvestitol, medicarpin, formononetin, daidzein	^29^, ^30^, ^31^, ^32^, ^33^, ^34^, ^35^, ^36^
Mixed propolis types	Two or three plant sources, e.g., aspen‐poplar propolis, *Cupressus*‐poplar propolis,^10^ Pacific‐*Mangifera indica* propolis^18^				

Although the chemical composition may vary, propolis from different origin usually demonstrates a considerable and comparable biological activity.^15^ Due to the discovery of a broad spectrum of biological activities associated with the utilization of this natural product, which include anti‐inflammatory, immunostimulant, antimicrobial, and antiviral properties, the interest in propolis has increased over the last few years unfolding new research and therapeutic horizons.^15^, ^37^, ^38^, ^39^

### 1.2 Viral infections

A virus is a nanometric infectious particle, or virion, consisting of genetic material (single‐ or double‐stranded DNA or RNA) contained in a protein coat, the capsid, and sometimes an outer lipidic envelope. Viruses replicate only inside living cells and can infect all known types of life forms. Viruses are currently classified by means of a unified taxonomy used in conjunction with the Baltimore classification system, which defines seven groups on the basis of the viral genome (single‐ or double‐stranded DNA, single‐stranded sense or antisense RNA, and double‐stranded RNA) and the mechanism of messenger RNA (mRNA) production (with or without the aid of reverse transcriptase).^40^

Historically, the first tenet of cell theory stated that living organisms consist of cells. However, viruses are sometimes considered noncellular living entities, even though they are not capable of autonomous reproduction without relying on cellular machinery to be copied. Whether a virus should be considered a living organism or a replicator at the edge of chemistry and life, it may be simply defined as “a piece of bad news wrapped in protein,” to quote the British biologist and Nobel Prize winner Sir Peter Medawar. Indeed, viruses are responsible for a wide variety of human diseases.

Viral infections provoke in animals an innate and an adaptive (humoral and cell‐mediated) immune response. The innate immune system detects and responds to pathogens in a nonspecific manner to defend the infected host cells. Viral recognition generally occurs in two different ways through the detection of specific molecular signatures: the recognition of pathogen‐associated molecular patterns (PAMPs), which are distinct molecular features of the viral particles, via pattern recognition receptors (PRRs), and the detection of cellular damage or stress induced by viral infection. As a result of PRR engagement, pro‐inflammatory cytokines, mainly downstream of NF‐κB activation, and type I interferons (IFNs) are induced. INF‐α and IFN‐β, collectively referred to as type I IFNs, are the major effector cytokines orchestrating the response of the host against viral infections.^41^ Additionally, type I IFNs link innate and adaptive immune responses, enhancing dendritic cell maturation, natural killer cell cytotoxicity, and differentiation of virus‐specific cytotoxic T lymphocytes.^42^ A second outcome is the inflammasome‐mediated activation of caspase‐1, which can cleave multiple substrates including pro‐interleukin (IL)−1β. Both PRR‐induced pathways can also initiate apoptosis in the attempt to prevent viral replication and spread.^43^, ^44^

One of the best‐characterized mechanisms for PRR activation is the recognition of viral nucleic acids, either viral genome or replication intermediates, which take place both at endosomal and cytosolic levels. Many toll‐like receptors (TLRs) able to detect PAMPs from several types of pathogens are expressed in endosomes and detect viral genomes upon endocytosis, triggering type I IFN expression.^45^, ^46^ TLR3, originally identified as a sensor of dsRNA viruses,^47^ activates dendritic cells after the phagocytosis of infected cells,^48^ and plays a role in protecting the central nervous system against herpes simplex virus infection.^49^, ^50^ TLR7/8 and TLR 9 recognize ssRNA and dsDNA viral genomes, respectively, and stimulate pro‐inflammatory cytokine and type I IFN expression.^51^, ^52^, ^53^ On the other hand, cytosolic sensors include families of structurally related receptors: RIG‐I‐like receptors, which are sensors of RNA and induce the expression of type I IFNs; AIM2‐like receptors, which are sensors of DNA and elicit inflammasome activation; NOD‐like receptors, which are sensors of viral PAMPs and virus‐induced cellular stress, and cause either IFN expression or inflammasome activation.^43^, ^54^

Cytokine production may be induced by the viral infection (primary cytokines) or be a consequence of the immune response (secondary cytokines). Although it is difficult to discriminate an intense pro‐inflammatory response due to severe infection from a dysregulated cytokine response,^55^ abnormally elevated levels of inflammatory cytokines are often referred to as “cytokine storm,” a condition that typically accompanies certain viral infections. For example, a significant number of deaths from influenza occurs due to cytokines despite early antiviral therapy. Given the role of inflammation in the pathogenesis of these diseases, cytokines may be one of the most critical targets for an immunomodulatory approach to viral infections.^56^ In this context, besides the direct role in virus clearance, also type I IFNs may be of pivotal importance exerting an anti‐inflammatory activity through the induction of IL‐10 production.^57^

Many natural products, including propolis, contain high amounts of polyphenolic compounds characterized by antioxidant properties and able to inhibit inflammatory cytokine production as well, mainly through the impairment of the transcriptional activity of NF‐κB.^58^ The use of such natural products might enhance endogenous host defenses in a nonspecific manner and modulate inflammatory processes, thus qualifying propolis as a possible prophylactic or therapeutic approach to infectious diseases.

**Figure 1:**
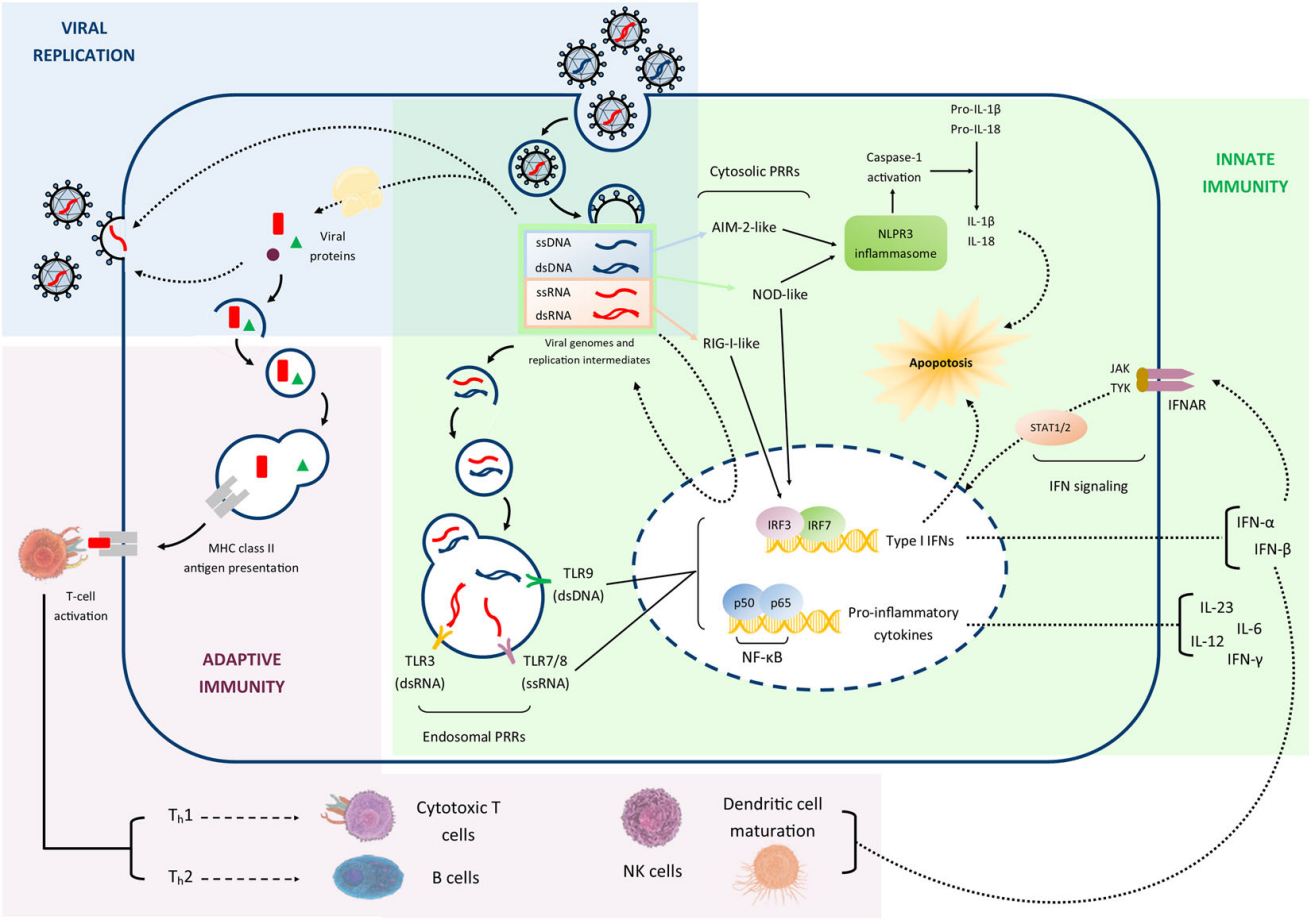
Schematic representation of the pathogenic mechanisms underlying viral infections, which represent the principal targets of propolis action. dsDNA, double‐stranded DNA; IFN, interferon; IL, interleukin; NK, natural killer; ssRNA, single‐stranded RNA [Color figure can be viewed at wileyonlinelibrary.com]

The principal targets of propolis action, represented by the pathogenic mechanisms described in this section, both related to the viral replication cycle and the innate and adaptive responses to viral signatures, are summarized in Figure 1.

## 2 METHODOLOGY

### 2.1 Research question

The aim of this review was to collect the existing in vitro, ex vivo, in vivo, and clinical evidence of the antiviral and immunomodulatory activities of propolis, to encourage the carrying out of rigorous clinical trials and help substantiate a rational therapeutic usage of this natural product against viral diseases, with a particular focus on the applications in respiratory affections. Whenever possible, a critical commentary of the collected evidence was offered, to unveil correlations between certain biological activities and specific propolis types or subtypes, dissecting antiviral and immunomodulatory mechanisms to clarify the possible modes of action and draw attention to the most promising products and lead compounds.

### 2.2 Data sources and search criteria

Articles were searched using Web of Science, Scopus, Embase, and PubMed databases, without any language restriction, using the following search terms: “propolis” AND (“antiviral” OR “virus” OR “influenza” OR “immunomodulation” OR “immunomodulatory” OR “immune” OR “inflammation” OR “respiratory” OR “airways”). The search (title, abstract, and keywords) reported 1050 items on Web of Science, 1251 on Scopus, 844 on Embase, and 730 on PubMed, indexed until July 2021. Duplicate articles were removed. In a second step following study selection, the references listed in the retrieved research articles and reviews were sifted through, to identify documents that might had eluded the primary search.

### 2.3 Eligibility criteria

Preclinical and clinical studies dealing with the effects of propolis produced by honeybees (*Apis mellifera *Linnaeus 1758) on immune mechanisms, respiratory system pathophysiology, and viruses relevant for human pathologies were considered eligible. Documents in languages different from English were included if they were published in Italian, or if an abstract in English was available and the abstract contained a sufficiently detailed description of research methods and results.

### 2.4 Study selection

Full titles and abstracts of the documents retrieved in the primary search were assessed for adherence to the eligibility criteria. Subsequently, full texts (except the cases in which the full text was unavailable with the means at our disposal) of the eligible articles were carefully read and checked for inclusion.

### 2.5 Data collection

Information relevant to the research question (in particular: type of propolis, study model and biological parameters, and statistically significant results) were deducted through the careful reading of abstracts and full texts of the articles. The methodological quality of the clinical trials reported in the antiviral activity section was assessed using the algorithm proposed by Jadad et al.^59^

## 3 ANTIVIRAL ACTIVITY

Many in vitro, in vivo, and clinical studies have highlighted the broad spectrum of antiviral activities which several types of propolis share against numerous viral families responsible for relevant human diseases. Still, in many cases, the nature of these effects is yet to be fully unraveled, and the understanding of their significance is complicated by the lack of consistency between clinical and preclinical studies.

Few works considered the overall clinical efficacy of propolis extracts against respiratory tract infections, a number of diseases caused in most cases by viruses with possible bacterial and fungal superinfections. In a case–control study carried out in pre‐school and school children, Crişan et al.^60^ evaluated the action of a proprietary aqueous flavonoid‐rich extract (NIVCRISOL) in acute inflammatory diseases of the upper respiratory tract, such as common cold, generally caused by rhinoviruses and, to a less extent, human coronaviruses, influenza viruses, or adenoviruses. The monitoring consisted of the recording of the incidence of acute rhinopharyngitis symptoms and in the periodical examination for the determination of viral burden. The results demonstrated a favorable effect of propolis local treatment in lowering the number of symptomatic cases and decreasing, and sometimes suppressing, the microbial flora of the upper airways.^60^ Since the authors specified neither the composition of the microbial flora nor the reduction of specific viral titers, an antiviral effect of propolis, albeit not unlikely, may only be hypothesized, along with a probable immunomodulatory and disease‐modifying activity. Cohen et al.^61^ conducted a randomized, double‐blind, and placebo‐controlled clinical trial to evaluate the safety and effectiveness of an herbal preparation (Chizukit) containing propolis extract (50 mg/ml), an extract of *Echinacea purpurea* (L.) Moench aerial parts and *Echinacea angustifolia* DC. roots, and vitamin C, in preventing respiratory tract infections in children. The study demonstrated that the treatment significantly decreased the number of children who experienced one or more respiratory tract illness episodes during the 12 weeks of the study, the total number of episodes, and the mean number of episodes per child (primary outcome). The total number of illness days and the duration of individual episodes were also significantly lower. Moreover, the treatment reduced the number of days of fever and the use of antipyretics, the incidence of rhinitis, and daytime and nighttime cough (secondary outcome).^61^ Once again, no mechanism against a specific viral species was reported, and besides, propolis was associated with other natural products, thus impeding the unequivocal attribution of a causal relationship. Szmeja et al.^62^ clinically tested the therapeutic value of flavonoid‐rich Canadian propolis in rhinovirus infections. The treatment shortened by 2.5‐fold the duration of the disease, with the regression of symptoms starting from the first day of therapy and the complete recovery within 3 days in the treated group, in contrast with an average of 4.80 days in the placebo group.^62^ Esposito et al.^63^ performed a randomized, double‐blind, placebo‐controlled clinical trial to evaluate the efficacy of an oral spray, formulated with a poplar‐type propolis proprietary extract (M.E.D.®) standardized through the evaluation of six markers (apigenin, chrysin, galangin, pinobanksin, pinocembrin, and quercetin) which represent the 25% of the total polyphenols indicated in the titration, on the remission of symptoms (sore throat, muffled dysphonia, and swelling and redness of the throat) associated with mild uncomplicated upper respiratory tract infections. The results demonstrated that after 3 days of treatment with propolis, 83% of subjects had remission of all symptoms, while 72% of subjects in the control group had at least one remaining symptom, indicating that propolis led to prompt resolution with an advance of 2 days.^63^ This evidence may be related to a newly demonstrated physical mechanism of propolis action consisting in the generation of an extensive “exclusion zone” water layer on propolis‐coated surfaces, which constitutes an effective barrier able to hinder microbial access to the potential site of infection and therefore inhibit viral entry.^64^

Finally, Di Pierro et al.^65^ investigated the role of another proprietary extract (Proposoma‐lisclatrato®), a mixture of phytosome and propolis co‐ground in a ratio 1:1, in an open‐label, retrospective, controlled clinical analysis, conducted in children with acute otitis media or viral pharyngitis, generally caused by paramyxoviruses, rhinoviruses or adenoviruses. The oral administration of propolis reduced the severity of symptoms, avoided the use of antipyretics and anti‐inflammatory drugs, and decreased the progression to tracheitis, bronchitis, and rhinosinusitis.^65^ In view of this, it may be easily inferred that the pharmaceutical formulation of propolis extracts used in clinical studies seems not a negligible factor. In their study, in fact, Drago et al.^66^ compared in vitro the antiviral activities of another proprietary product, Actichelated® propolis (a blend of active substance, carrier, and biocatalyst subjected to mechano‐chemical activation), and of a hydroalcoholic extract of propolis collected in Argentina and Uruguay, showing that the active antiviral concentrations of Actichelated® propolis against different strains of adenoviruses, influenza viruses, parainfluenza viruses, and herpes simplex virus type 1 were at least ten times lower than those of the hydroalcoholic extract.

As discussed above, propolis exerts a clinically plausible antiviral activity, despite the existence of confounding factors such as the type of propolis extract, the route of administration, or the pharmaceutical formulation. However, due to the variability in the etiology of these diseases, only a favorable role of propolis treatment could be demonstrated, without any elucidation of the effects against specific viral species. Nonetheless, a common thread among all these clinical studies is the use of flavonoid‐rich propolis, but the contribution of an immunomodulatory and anti‐inflammatory activity to the overall effect may not be excluded.

Many in vitro studies have tried to define the general mechanisms of action, which are not completely understood, at a cellular and molecular level. One of the most critical steps in antiviral innate immunity, for example, is the recognition of viral genome by PRRs which results, among other things, in the induction of type I IFNs and, consequently, of antiviral IFN‐inducible genes. Hayakari et al.^67^ have demonstrated in a model of viral‐like infection that pretreatment of A549 human alveolar epithelial cells with green Brazilian propolis could inhibit poly I:C‐induced IFN‐β mRNA and protein expression in a concentration‐dependent manner and enhance the mRNA expression of antiviral factors. Hence, propolis could increase the expression of antiviral effectors also through IFN‐independent pathways. Moreover, propolis prevented poly I:C‐induced mRNA expression of the pro‐inflammatory chemokines IL‐8 and CCL5, thus hindering the chemotaxis of polymorphonuclear leukocytes exposed to cell‐conditioned medium.^67^ These results once again highlight the importance of the immunomodulatory and anti‐inflammatory activities, which coexist in propolis with the merely antiviral ones.

In the following paragraphs, the known direct effects of propolis on individual viral species are presented. For ease of treatment, viral species are divided according to the Baltimore classification group and taxonomic family. Table 2 lists details and results of the clinical studies reporting evidence of propolis antiviral activities.

**Table 2:** Clinical studies assessing the antiviral properties of propolis and propolis extracts

	Virus (disease)	Propolis type/extract	Oxford quality scoring system^a^	Intervention		Control		Statistically significant results
				Sample size	Propolis concentration	Sample size	Type of control	
Hoheisel et al.^68^	HSV‐1 (recurrent herpes labialis)	ACF®	5	33	3% ointment	35	Placebo ointment	The rate of lesion healing was faster among the patients treated with propolis ointment (mean 6.24 days) compared to those treated with placebo (mean 9.77 days) (*p* < 0.00001). The patients who applied propolis ointment were without pain considerably earlier than those who applied the placebo ointment (*p* = 0.00671).
Holcová and Hladíková ^69^	HSV‐1 (herpes labialis)	GH 2002	5	48	0.1% lip balm	n/a	n/a	The best efficacy with shortest healing time and good tolerability were observed with the 0.5% concentration (3.4 and 5.4 days in the 50th and 90th percentile, respectively; *p* = 0.008 vs. 1% and 0.09 vs. 0.1%). All concentrations achieved therapeutic results in comparison with baseline values (*p* < 0.0005) for all secondary parameters (local pain, itching, burning, tension, swelling, and tolerability) as early as Day 2/3.
				50	0.5% lip balm			
				52	1% lip balm			
Arenberger et al.^70^	HSV‐1 (herpes labialis papular/erythematous stage)	GH 2002	3	189	0.5% lip balm	190	5% acyclovir cream	The desired clinical outcome was reached after a median of 4 days with propolis and 5 days with acyclovir (*p* < 0.0001). Significant differences in favor propolis were observed for all secondary parameters. No allergic reactions, local irritations, or other adverse events were registered.
Jautová et al.^71^	HSV‐1 (herpes labialis vesicular stage)	GH 2002	5	199	0.5% lip cream	198	5% acyclovir cream	The primary clinical endpoint (difference in time between groups to complete encrustation or epithelization of the lesions) was reached after a median of 3 days with propolis and 4 days with acyclovir (*p* < 0.0001). Significant differences in favor of propolis were observed for all secondary parameters. No allergic reactions, local irritations or other adverse events occurred.
Vynograd et al.^72^	HSV‐2 (recurrent genital infection)	ACF®	2	30	3%	30	5% acyclovir ointment	On Day 10, 24 out of 30 patients in the propolis group had healed. In the acyclovir group 14 out of 30, and in the placebo group 12 out of 30 had healed (*p* = 0.0015). The healing process appeared to be faster in the propolis group: 15 individuals had crusted lesions on Day 3 compared to 8 individuals in the acyclovir group and none in the placebo group (*p* = 0.0006).
						30	pH‐neutral placebo ointment	
Tomanová et al.^73^	VZV (herpes zoster)	GH 2002	1 (open‐label trial)	33	0.5% lotion + oral acyclovir	27	Placebo lotion + oral acyclovir	Improvement of pain was better and quicker in the propolis lotion group (*p* < 0.001). At least 50% of propolis‐treated patients were lesion‐free on Day 14, versus Day 28 in the control group (*p* = 0.013). The formation of new vesicles was also suppressed (*p* < 0.001). No allergic reactions, skin irritations, or other adverse events were observed.
Crişan et al.^60^	Respiratory tract viruses	NIVCRISOL (aqueous extract)	0 (case‐control study)	n/a	n/a	n/a	n/a	Favorable effects of the treatment, expressed by lowering of the number of cases with acute or chronic symptoms, and decrease and sometimes suppression of the viral‐microbial flora carriage of the upper airways. (Unknown statistical significance)
Cohen et al.^61^	Respiratory tract viruses	n/a (propolis, *Echinacea* spp., and vitamin C elixir)	5	160	250–375 mg of propolis twice a day for 12 weeks	168	Identical placebo	After 12 weeks, a significant reduction of illnesses was observed in the treated group with regard to the number of illness episodes (138 vs. 308; 55% reduction), number of episodes per child (0.9 ± 1.1 vs. 1.8 ± 1.3; 50% reduction, *p* < 0.001), and number of days with fever per child (2.1 ± 2.9 vs. 5.4 ± 4.4; 62% reduction, *p* < 0.001). The total number of illness days and the duration of individual episodes were also significantly lower in the treated group. Adverse drug reactions were rare, mild, and transient.
Szmeja et al.^62^	Rhinovirus	Canadian propolis	n/a	n/a	n/a	n/a	n/a	The observed therapeutic effect was the shortening of the disease duration. Complete recovery occurred in 1 day in 5 patients, in 2 days in 16 patients, and in 3 days in 3 patients. The placebo group fully recovered in a mean of 4.80 days. In the therapeutic group the symptoms lasted 2.5 time shorter than in placebo one. (Unknown statistical significance)
Esposito et al.^63^	Respiratory tract viruses	M.E.D.®	5	58	Propolis oral spray (12–24 mg polyphenols/die for 5 days)	64	Identical placebo	After 3 days of treatment, the oral application of propolis was significantly associated with the remission of all symptoms (*p* < 0.001) and individual symptoms, such as sore throat (*p* < 0.001), swelling and redness of throat (*p* < 0.001), and muffled dysphonia (*p* = 0.022); 83% of subjects treated with propolis oral spray had remission of symptoms, while 72% of subjects in the placebo group had at least one remaining symptom.
Di Pierro et al.^65^	Respiratory tract viruses (acute otitis media, viral pharyngitis)	Proposoma‐lisclatrato®	0 (open‐label, retrospective study)	28	200 mg every 6 h for a maximum of 72 h	28	No treatment	Propolis supplementation for 72 h lessened the severity of acute otitis media and viral pharyngitis, reduced the use of antipyretics and anti‐inflammatory drugs, and decreased the rate of evolution to tracheitis, bronchitis, and rhinosinusitis (*p* < 0.05).
Iljazović et al.^74^	HPV (genital lesions)	n/a	0	35	Interferon + 5% propolis pessaries + B vitamin complex	20	Other therapies	After three months HPV infection was still present in more than 90% of the patients in the control group. In the treated group HPV infection had disappeared in 71.42% of the patients after 3 months and in 100% of the patients after 6 months.
Zedan et al.^75^	HPV (warts)	n/a	2	45	Pure propolis 500 mg/die *per os*	50	Placebo capsules	After 3 months propolis was effective in treating plane and common warts in 75% (*p* < 0.05) and 73% (*p* < 0.01) of patients, respectively.
Soroy et al.^76^	DENV (dengue hemorrhagic fever)	Propoelix™ (blend of poplar and *Baccharis* spp. propolis)	5	31	400 mg/die for 7 days	32	Placebo	Platelet counts in the treated group showed a faster recovery by Day 6 (*p* = 0.042) and Day 7 (*p* = 0.006). Patients treated with propolis had a greater decline in TNF‐α levels on Day 7 compared with patients in the placebo group (*p* = 0.018). The length of hospitalization was also shorter (*p* = 0.012).
Silveira et al.^77^	SARS‐CoV‐2 (COVID‐19)	EPP‐AF®	3 (open‐label trial)	40	400 mg/die for 7 days plus standard care	42	Standard care (supplemental oxygen, noninvasive or invasive ventilation, corticosteroids, antibiotics and/or antiviral agents, vasopressor support, renal‐replacement therapy, intra‐aortic balloon pump and extracorporeal membrane oxygenation, as necessary)	At 28‐day follow‐up the length of hospitalization was significantly lower in groups receiving propolis than in the control group, with a mean difference of −3.03 days (median 7 vs. 12 days; *p* = 0.049) for the 400 mg/die group and −3.88 days (median 6 vs. 12 days; *p* = 0.009) for the 800 mg/die group. Patients treated with 800 mg/die of propolis had a significantly lower rate of acute kidney injury than the control group (*p* = 0.048).
				42	800 mg/die for 7 days plus standard care			

Abbreviations: COVID‐19, coronavirus disease 2019;SARS‐CoV‐2, severe acute respiratory syndrome coronavirus 2.

^a^ The methodological quality of the clinical trials reported in this table was assessed using the algorithm proposed by Jadad et al. 59 based on the effectiveness of blinding. A score between 0 (very poor) and 5 (rigorous) is allocated to each trial.

### 3.1 Double‐stranded DNA viruses

#### 3.1.1 Herpesviridae

Epstein‐Barr virus (EBV) is a human herpesvirus responsible for infectious mononucleosis, as well as various nonmalignant, premalignant, and malignant lymphoproliferative diseases. Chang et al.^78^ investigated in vitro the effect of moronic acid, a triterpenoid isolated from southern Brazilian propolis, on EBV. The treatment of an EBV‐bearing Burkitt's lymphoma cell line (P3HR1) with moronic acid inhibited the expression of viral key transcription factors during the lytic cycle. In particular, it interfered with the transactivation functions of Rta viral protein, impairing the production of functional EBV virions.^78^

Herpes simplex virus type 1 (HSV‐1) and 2 (HSV‐2) are the etiological agents of herpes labialis and genital herpes, respectively. Varicella‐zoster virus (VZV) causes varicella, a disease usually affecting children, and herpes zoster, which commonly affects adults. Acyclovir, a nucleoside analog able to arrest viral DNA synthesis, is the principal conventional drug used for the treatment of HSV‐1/2 and VZV infections. However, adverse effects and the emergence of drug‐resistant viral strains demand novel therapeutic agents. When HSV‐1 viral particles were pretreated with propolis hydroalcoholic (70% ethanol) extract for 1–3 h before being added to HEp‐2 cells, significant antiviral efficacy was observed at a concentration of 10 μg/ml, alone or in combination with acyclovir, with the greatest decrease in viral titers within the first hour.^79^ In another study, the antiviral activity of Turkish propolis collected in the Hatay province was assayed against HSV‐1 and HSV‐2. Viral replication was significantly suppressed in the presence of propolis, as shown by the decrease in viral titers, and the effect was more massive and rapid against HSV‐1. Moreover, the combination of propolis and acyclovir displayed a synergistic effect, proving to be more effective than acyclovir alone.^80^ These results show the ability of propolis to impair, at least in vitro, the replication of herpes simplex virus through direct antiviral activities.

Shimizu et al.^81^ evaluated three ethanolic extracts of Brazilian propolis collected in various States and with different botanical origin, namely *Baccharis dracunculifolia* DC., *Baccharis erioclada* DC., and *Myrceugenia euosma* (O. Berg) D. Legrand. The oral administration of propolis extracts to BALB/c mice inoculated with HSV‐1 virus delayed the development and progression of herpetic skin lesions in the early phases of infection. Propolis from *B. dracunculifolia*, that is, green propolis, was significantly effective in reducing viral titers in vivo, in the skin and brain of infected mice, and in vitro, in a plaque reduction assay. Propolis from *M. euosma*, of which moronic acid is a characteristic constituent, was active in reducing viral titers in vivo only in the brain but was inactive in the in vitro assay. Nonetheless, it enhanced delayed‐type hypersensitivity to HSV‐1 antigens and increased IFN‐γ production from splenocytes of HSV‐1‐infected mice, suggesting the presence of components active in vivo after oral administration. Propolis from *B. erioclada*, despite being active in vitro, had no significant effect on viral titers in either skin or brain but significantly enhanced delayed‐type hypersensitivity in infected mice and elevated IFN‐γ production in splenocytes in vitro.^81^ In turn, the oral administration of brown Brazilian propolis hydroalcoholic (70% ethanol) extract protected female BALB/c mice against acute vaginal lesions caused by HSV‐2. The treatment reduced extra‐vaginal lesions (swelling, edema, and inflammation) and histopathological alterations (leukocyte infiltration) induced by HSV‐2 infection. Moreover, propolis extract promoted a protective effect by acting on inflammatory and oxidative processes.^82^ Altogether, these findings indicate that several Brazilian propolis subtypes seem effective in vivo against both HSV‐1 and HSV‐2 infection, with slight but significant differences in their modes of action which were not extensively investigated. In search of molecular mechanisms of action, Huleihel and Isanu showed that 0.5% propolis extract determined 50% inhibition of HSV‐1 infection in Vero cells, providing indirect evidence of an interaction between propolis extract and cell surface; however, no evidence of a direct interaction with viral particles could be found. Based on these results, the antiviral efficacy of propolis extract against HSV‐1 infection may be attributed, at least in part, to the prevention of virus absorption onto the host cells.^83^ However, the authors did not state the origin of the propolis used in the study, therefore these conclusions may not apply to Brazilian propolis.

A few in vitro and in vivo studies have been conducted to assess the anti‐herpetic potential of a proprietary hydroalcoholic (90% ethanol) extract (GH 2002), obtained from crude propolis collected in central Europe, highly purified and freed from pollen, beeswax, and resins. The resulting extract is enriched in flavonoids, polyphenols, and phenylcarboxylic acids. IC_50_ for VZV plaque formation was determined at 64 µg/ml, while in viral suspension tests infectivity was significantly reduced by 93.9%, accounting for a direct concentration‐dependent antiviral activity with an efficacy comparable to acyclovir. Anti‐VZV activity was mainly exerted when viruses were pretreated with propolis before infection, thus indicating an unspecific interaction between viruses and propolis.^37^ Nolkemper et al.^84^ compared GH 2002 with an aqueous extract, equally rich in phenylcarboxylic acids but displaying a very low content of flavonoids and lacking galangin and quercetin. The cytotoxic and anti‐herpetic effects of the extracts were assessed in vitro against HSV‐2 in RC‐37 cell. IC_50_ of aqueous and GH 2002 extracts for HSV‐2 plaque formation were determined at 5 µg/ml and 4 µg/ml, respectively. Both extracts showed high antiviral activity against HSV‐2 in viral suspension tests, with >99% reduction of infectivity, exerting a direct concentration‐ and time‐dependent activity when viruses were pretreated before incubation with cells.^84^ These results demonstrate that flavonoids produce a synergistic, or at least additive, effect together with phenylcarboxylic acids and other phenolic compounds, achieving lower effective concentrations. Nonetheless, flavonoids appear not to be primarily responsible for the activity. Different polyphenols, flavonoids, and phenylcarboxylic acids have been identified as major constituents of the above‐mentioned aqueous and GH 2002 extracts, including caffeic acid, *p*‐coumaric acid, benzoic acid, galangin, pinocembrin, and chrysin. Therefore, the antiviral activity of the extracts and their isolated compounds was also evaluated against HSV‐1 in RC‐37 cells. IC_50_ of aqueous and GH 2002 extracts for HSV‐1 plaque formation were determined at 4 µg/ml and 3.5 µg/ml, respectively, demonstrating also in this case a higher activity for extracts richer in flavonoids. Both extracts showed high antiviral activity against HSV‐1 in viral suspension tests, with >98% reduction of infectivity. Once again, the anti‐herpetic activity was only observed when viruses were pretreated before infection. Among the isolated compounds, only galangin and chrysin (a flavonol and a flavone, respectively) displayed some antiviral activity,^85^ not clearly explaining the activity of whole propolis extracts and the fact that propolis is almost equally effective in spite of the presence of high or low concentrations of flavonoid. Moreover, these results are partially in contrast with the work of Debiaggi et al.^86^ in which also kaempferol, as well as chrysin, proved to cause a concentration‐dependent reduction of intracellular replication of herpesvirus strains. However, virus infectivity was not significantly reduced. On the other hand, galangin had no effect on either the infectivity or replication, whereas quercetin was able to reduce infectivity and intracellular replication, but only at the highest concentration tested.^86^ The extracts exhibited significantly higher activity and selectivity indices in any case, showing that the observed effects cannot be reduced to the effect of single components, but synergisms play a prominent role. This evidence might find confirmation in the work of Amoros et al.^87^ in which, besides the fact that flavonols (e.g., galangin, quercetin, rutin, and kaempferol) resulted to be more active against HSV‐1 than flavones (e.g., chrysin, apigenin, and acacetin), binary flavone‐flavonol combinations proved to be more effective than individual compounds, and such a synergistic effect could explain why total propolis extracts are more active than isolated components.^87^ Demir et al.^88^ have recently investigated in vitro the antiviral activity against HSV‐1 and HSV‐2 of M.E.D.® propolis extract formulated in different vehicles (propylene glycol, ethanol, glycerol, and soya oil). The determination of the selectivity indices demonstrated for the glycolic propolis extract a greater antiviral activity than acyclovir against both HSV‐1 and HSV‐2, whereas the ethanolic and soya oil extracts were found to be more active than acyclovir only against HSV‐2.^88^ Notably, the extracts that showed the most promising activity in this study are all cosmetic ingredients which could advantageously be exploited in the formulation of products for topical application.

The efficacy of propolis against herpes viruses has been evaluated also in humans and a consistent number of clinical studies have been conducted on formulations containing GH 2002 extract. Holcová and Hladíková^69^ evaluated the efficacy and tolerability of a lip balm against herpes labialis in a double‐blind, randomized, three‐arm dose‐finding study with three concentrations of GH 2002 (0.1%, 0.5%, and 1%). The best results were obtained with 0.5% formulation, which allowed the shortest healing time (3.4 and 5.4 days in the 50th and 90th percentile, respectively), good tolerability, and significant therapeutic results for all the secondary parameters (local pain, itching, burning, tension, and swelling).^69^ Then, Arenberger et al.^70^ conducted a single‐blind, randomized study to compare a lip balm containing the selected concentration of 0.5% GH 2002 and 5% acyclovir cream, focusing their attention on patients with herpes labialis in the papular/erythematous stage. The primary endpoint (complete encrustation or epithelization of the lesions) was reached after 4 days with propolis and 5 days with acyclovir. Significant differences in favor of propolis treatment were also observed for the secondary parameters (pain, burning, itching, tension, and swelling). No allergic reactions, local irritations, or other adverse events were observed.^70^ A following double‐blind, randomized, multicentre trial with 400 patients evaluated the effect of 0.5% GH 2002 lip cream, compared with 5% acyclovir, for the treatment of the vesicular stage of herpes labialis. This time, the primary endpoint was reached after 3 days with propolis and 4 days with acyclovir, and, also in this case, significant differences in favor of propolis treatment were observed for the secondary parameters.^71^ Altogether, these results show the overall efficacy of the topical application of GH 2002 propolis extract against herpes labialis, irrespective of the stage of the disease, susbstantiating the activity against HSV‐1 previously observed in vitro. Tomanová et al.,^73^ based on the results of a previous uncontrolled trial by Holcová and Hladíková,^89^ investigated in an open controlled trial the efficacy of a lotion containing 0.5% GH 2002 propolis extract as a supportive add‐on therapy to oral acyclovir treatment against herpes zoster, caused by VZV. The treatment with propolis lotion improved pain starting from Day 3 to 4. Herpetic lesions healed significantly quicker with propolis treatment: at least half of patients receiving propolis lotion were free of lesions at Day 14, whereas a similar outcome was reached in the control group only at Day 28. Formation of new vesicles was significantly suppressed, and skin tolerability was excellent. No allergic reactions, skin irritations, or other adverse events were observed.^73^

Other studies were conducted using different proprietary extracts. A single‐blind, randomized, controlled, multi‐center study was undertaken to evaluate the efficacy of Canadian propolis ointment compared with acyclovir in men and women with recurrent chronic genital HSV‐2 infection. Propolis was collected in a region rich in *Populus* spp. trees and extracted with 95% ethanol. The proprietary extract, designated ACF® (Antiviral Complex of Flavonoids), was not standardized to a certain component. The healing process seemed to be faster in the group receiving the propolis ointment, which appeared to be more efficacious than both placebo or acyclovir in the resolution of genital herpetic lesions and of local symptoms. Moreover, the incidence of bacterial superinfection was reduced by 55%.^72^ Bankova et al.^90^ analyzed the ACF® propolis extract with the aim of determining its chemical composition to understand the plant origin and the possible connections between the identified compounds and the antiviral activity. GC‐MS characterization revealed components deriving from the resins of two different species of poplar, *P. tremuloides* Michx. (sec. *Populus*) and *P. balsamifera* L. (sec. *Tacamahaca*), with high content of benzoic acid, *p*‐coumaric acid, benzyl *p*‐coumarate, and dihydrochalcones (pinocembrin chalcone and pinostrobin chalcone). The chemical composition appeared standardized between different extract samples and was also reproduced in a sample of topical ointment. In vitro, the extract showed a pronounced virucidal effect when HSV‐1 and HSV‐2 were incubated with propolis extract before infection and interfered with HSV‐1 adsorption on the host cell surface.^90^ Subsequently, Hoheisel evaluated in a randomized, double‐blind, controlled clinical trial the efficacy of an ointment containing 3% ACF® extract against recurrent herpes labialis. Patients treated with propolis ointment showed a significantly faster healing rate and were without pain considerably earlier. In particular, treated patients appeared to improve faster in the early days of ointment application, even though no differences were found in the size of lesions.^68^ An extensive analysis of similarities and differences between the proprietary extracts used in clinical evaluations would be of great help to achieve a full understanding of the peculiarities needed to obtain the therapeutic efficacy.

The results of in vitro studies have shown a prominent direct activity on viral particles and synergistic effects with acyclovir, possibly through different mechanisms of action (e.g., direct virucidal activity, inhibition of viral internalization/replication/shedding), suggesting the usefulness of propolis as an add on therapy in combination with antiviral drugs. The clinical studies have confirmed the value of propolis‐containing preparations, either alone or co‐administered with acyclovir to reduce doses and adverse effects. Altogether, the existing evidence on propolis anti‐herpetic effects suggests that formulations containing flavonoid‐rich propolis extracts might shorten the temporal course of the disease, thus being suitable for topical application in recurrent herpetic infections. Nevertheless, the absence of clinical confirmation of other promising preclinical results regarding different propolis types and the oral route of an administration still claims further exploration.

#### 3.1.2 Papillomaviridae

Human papilloma virus (HPV) may cause persistent infections that result in warts or pre‐cancerous oral and genital lesions. The aim of the study by Iljazović et al.^74^ was to clinically evaluate the efficacy of an association between interferon and a propolis herbal product in the treatment of genital HPV infection. Fifty‐five HPV positive women were enrolled in the study and randomly assigned to control group (other therapeutic options, e.g., laser, cryotherapy, and podophyllin) or treatment group (interferon pessaries + pessaries containing 5% propolis extract, *Aloe vera* (L.) Burm.f. juice, *Echinacea purpurea* (L.) Moench and *Calendula officinalis* L.). After 3 months, HPV infection was still present in more than 90% of the subjects in the control group, while had disappeared in 71.42% of the patients in the treatment group and in 100% after 6 months.^74^ In this study, however, propolis was formulated with other natural products and associated with interferon, making it impossible to dissect its individual contribution to therapeutic success. Zedan et al.^75^ conducted an open‐label, single‐blinded, randomized, placebo‐controlled clinical trial to investigate the effect of the dietary supplementation of propolis as an alternative treatment for cutaneous warts. In the case of flat and common warts, propolis treatment seemed effective in 75% and 73% of cases, respectively.^75^ In consideration of the oral administration, an immunomodulatory and disease‐modifying effect, rather than a direct antiviral one, cannot be excluded. To date, due to the absence of in vitro studies corroborating possible direct antiviral mechanisms, clinical evidence is insufficient to establish propolis efficacy against HPV.

#### 3.1.3 Poxviridae

*Parapoxvirus* is a genus of viruses, mainly zoonotic, responsible for causing orf (ecthyma contagiosum), a viral exanthem, in humans. Zeedan et al.^91^ evaluated the efficacy of ethanolic and aqueous Egyptian propolis extracts, administrated via subcutaneous and intradermal injections in albino rats inoculated with parapoxvirus. Propolis determined a nearly 2–3 log decrease of infectivity titers in vitro. In vivo, noninfected animals receiving propolis showed a slight suppression of TNF‐α and IFN‐γ levels when compared to controls, whereas the cytokine production appeared strongly stimulated in infected rats treated with propolis. The histopathological analysis revealed acute necrotic hepatitis accompanied with disseminated intravascular coagulopathy, which is a pathognomonic process, in infected animals. Conversely, rats treated with propolis appeared normal or presented only mild lesions.^91^ The in vitro evidence supports the hypothesis of a direct antiviral activity that may be relevant also in vivo, a context in which, however, it is accompanied by evident immunomodulatory effects.

### 3.2 Positive‐sense single‐stranded RNA viruses

#### 3.2.1 Flaviviridae

Dengue hemorrhagic fever is a mosquito‐borne zoonosis caused by dengue virus strains (DENV). Soroy et al.^76^ evaluated the effectiveness of a water‐soluble proprietary extract (Propoelix™, a blend of poplar and *Baccharis* spp. propolis), rich in caffeic acid phenethyl ester (CAPE) and flavonoids, on the clinical course of patients with dengue hemorrhagic fever. The results of this double‐blind, randomized, placebo‐controlled trial showed a trend toward faster recovery in platelet counts of treated patients, who had a significantly shorter length of hospitalization. Moreover, treated patients showed also a significant decline in TNF‐α levels, consistent with a possible anti‐inflammatory and overall disease‐modifying effect of propolis extract.^76^ This study is supported neither by the determination of viral titers nor by any known mechanism of propolis action against DENV, and a mere immunomodulatory effect on the host should not be excluded.

#### 3.2.2 Picornaviridae

Human rhinoviruses, assigned to the genus *Enterovirus* and categorized in three species (A, B, and C), are the major cause of upper respiratory tract infections, accounting for more than 50% of common colds. Rhinoviruses are also associated with severe lower respiratory tract symptoms and exacerbations of chronic pulmonary diseases, as well as fatal pneumonia in elderly and immunocompromised adults. Kwon et al.^92^ assayed in vitro the efficacy of Brazilian propolis hydroalcoholic (80% ethanol) extract, its fractions, and some isolated compounds against human rhinoviruses in HeLa cells. Based on IC_50_ values, the chloroform‐ and ethyl acetate‐soluble fractions, enriched in flavonoids and other phenolic compounds, showed the highest antiviral activity, followed by the hexane‐soluble fraction. The bio‐guided fractionation led to the identification of two active compounds, kaempferol (IC_50 _= 7.3–12.9 µM) and *p*‐coumaric acid (IC_50 _= 371.2–604.3 µM). Kaempferol, and other known propolis components (chrysin, galangin, quercetin, luteolin, fisetin, caffeic acid, ferulic acid, and acacetin), exhibited a greater antiviral activity than ribavirin and high selectivity. These results suggest that kaempferol and *p*‐coumaric acid may hinder the viral entry in the early stage of the infection, abating viral replication. In addition, ICAM‐1 mRNA and IL‐6 expression levels were significantly reduced,^92^ indicating the anti‐inflammatory potential of these compounds. Since rhinoviruses are among the main etiological agents of upper respiratory tract infections, one of the most common traditional applications of propolis, more targeted studies should be undertaken on the one hand in vitro, to clarify the antiviral molecular mechanisms of different propolis types, on the other at a clinical level, specifically considering pathogens rather than the clinical presentation of the disease, which may often be ambiguous.

The antiviral activities of Brazilian propolis hydroalcoholic (70% ethanol) extract and isolated compounds (caffeic and cinnamic acids) were evaluated against the replication in HEp‐2 cells of poliovirus type 1 (PV‐1), an enterovirus which is the causative agent of poliomyelitis. The effect on PV‐1 replication was determined at three different stages: cell pretreatment, simultaneous treatment, and postinfection treatment. The highest antiviral activity and decrease in viral RNA yields, in cell lysates as well as in supernatants, were observed when propolis extract and PV‐1 were added simultaneously to cell cultures. In pre‐treatments, which involved removal of the extract before infection, viral entry was higher, whereas in post‐treatments it was viral RNA quantification to be higher. Isolated compounds, caffeic and cinnamic acids, showed a lower antiviral activity if compared to propolis extract, thus suggesting once again their only partial involvement in the antiviral effects when considered individually. According to the authors, propolis might partially block the viral entry within cells, affect the steps of viral replication into cells, or degrade RNA before the virus entry into cells or after virus shedding to the supernatant. However, further investigations are still needed to unravel these mechanisms of action.^93^

#### 3.2.3 Leviviridae

MS2 virus is a bacteriophage that infects *Enterobacteriaceae*. Nevertheless, MS2 virus possesses structural and genetic characteristics similar to those of noroviruses, which belong to the family of *Caliciviridae* and represent one of the most common causes of human gastroenteritis. The antiviral effects of green and red Brazilian propolis hydroalcoholic (80% ethanol) extracts were evaluated against the norovirus surrogate MS2. The extracts showed antiviral effects that were dependent on propolis type and extraction process. Ultrasound‐extracted red propolis was the most effective, showing concentration‐ and time‐dependent activity, followed by red propolis extract obtained through maceration. In contrast, green propolis extracts showed inferior activity against the bacteriophage. Once again, the extract obtained with the aid of ultrasounds performed better than the macerated one.^94^ Red Brazilian propolis, as previously stated, is notoriously richer in flavonoids than green propolis, which is in turn characterized by the presence of prenylated phenolic acids. This evidence remarks on the significant contribution of flavonoids to antiviral activities. Tang et al.^95^ evaluated in vitro the inhibitory effect of aqueous and ethanolic propolis extracts on norovirus surrogate MS2 and murine norovirus. Increasing concentrations of the ethanolic extract caused a significant reduction of viral titers (3.75 and 6.93 log reduction for MS2 and murine norovirus, respectively, at 500 μg/ml), with a maximum effect in the first 40 min. Further analysis by transmission electron microscopy demonstrated that propolis directly acted on viral particles to prevent viral entry into the host cells.^95^

#### 3.2.4 Coronaviridae

To date, seven human coronaviruses, belonging to the subfamily *Orthocoronavirinae* and descending from the bat viral gene pool, are known and classified into two genera: *Alphacoronavirus* (including 229E and NL63) and *Betacoronavirus* (including OC43, HKU1, MERS‐CoV, SARS‐CoV, and SARS‐CoV‐2). Coronaviruses typically cause mild respiratory infections, such as the common cold, however, they may also be the cause of potentially lethal respiratory syndromes and epidemic or pandemic outbreaks, such as SARS (Severe Acute Respiratory Syndrome, including COVID‐19) and MERS (Middle East Respiratory Syndrome).

Silveira et al.^77^ conducted an open‐label, randomized, controlled trial on hospitalized adult COVID‐19 patients to assess the effectiveness of a 7‐day treatment with Propomax® capsules (400 and 800 mg/die, formulated with the standardized green Brazilian propolis extract EPP‐AF®) in conjunction with standard care. The length of hospitalization was significantly reduced in propolis‐treated groups, although propolis did not significantly affect the need for oxygen supplementation. Nevertheless, patients treated with propolis tended to have a reduced need for invasive oxygen therapy. In addition, in patients treated with the higher dose (800 mg/die), a lower rate of acute kidney injury, a common complication of the disease associated with a poor prognosis, was observed.^77^ The authors were not able to explicate the mechanisms behind the beneficial effects on COVID‐19 patients, however, the antioxidant, immunomodulatory and anti‐inflammatory properties of propolis could explain the reduction of the disease impact.

### 3.3 Negative‐sense single‐stranded RNA viruses

#### 3.3.1 Orthomyxoviridae

Many subtypes of influenza A virus are the etiological agents of epidemic and pandemic influenza. Kujumgiev et al.^15^ analyzed propolis hydroalcoholic (70% ethanol) extracts from samples of different geographical origins (Bulgaria, Albania, Mongolia, Egypt, Brazil, and Canary Islands) to determine in vitro their antiviral activity against avian influenza A/chicken/Germany/27, strain Weybridge (H7N7) virus, a subtype able to infect also humans. Most of the samples showed antiviral activity with similar efficacy in CEF cells, in spite of the great differences in chemical composition. Therefore, since different molecular combinations exert similar biological activities, propolis may have general pharmacological value as a natural mixture and not as a source of isolated compounds,^15^ reflecting the evidence that whole extract activity appears to be greater if compared to individual components. Nonetheless, Serkedjieva et al.^96^ evaluated in vitro the antiviral activity of six synthetic substances, esters of substituted cinnamic acids, which were identical with or analogous to propolis components found in the etheric fraction of a methanolic extract. The authors found that synthetic isopentyl ferulate, a close analog of isopent‐3‐enyl ferulate present in propolis, significantly suppressed the replication of influenza A/Hong Kong/1/68 (H3N2) virus and the production of viral hemagglutinins in vitro and in ovo. The compound was much less active against influenza A/PR/8/34 (H1N1) virus. Effective concentration ranges of isopentyl ferulate and propolis extract etheric fraction were almost equal, and the maximal effect was observed when the substance was present in the culture medium during the whole infectious process.^96^ These findings demonstrate that the antiviral activity of the fraction in a study can be unambiguously attributed to the aforementioned phenolic acid ester, arousing interest also for the research of new effective antiviral lead compounds in propolis. An aqueous propolis extract, rutin, and a rutin/quercetin combination were assayed in mice infected with influenza A/PR/8/34 (H1N1) virus. When propolis extract was administrated intranasally before virus inoculation, a reduction in viral hemagglutinin titers in lungs was observed, but no reduction in mortality or increase in survival times could be seen. However, when propolis was administrated after infection, the reduction in hemagglutinin titers was accompanied by a slight decrease in mortality. Rutin and rutin/quercetin combination were ineffective and actually increased both hemagglutinin titers and mortality.^97^ Despite the unclear significance of these findings, it is evident that propolis extract displays a biological activity not paralleled by its isolated components. Moreover, the fact that a decrease in mortality was only observed when propolis was administered after infection possibly indicates an immunomodulatory, as well as, antiviral effect. Governa et al.^98^ evaluated the pharmacological properties of a chemically characterized sample of European poplar propolis in which galangin and pinocembrin were the most representative flavonoid markers. CAPE was also present in high concentrations. A direct anti‐influenza activity was not clearly seen on MCDK cells compared to oseltamivir, in fact, antiviral activity emerged only at cytotoxic concentrations without any selectivity against influenza A/PR/8/34 (H1N1) virus. However, a plausible role for propolis in the inhibition of neuraminidase activity related to virus entry and shedding was confirmed, at least in part, with an enzymatic assay.^98^

A few studies considered propolis samples collected in Brazil. In an in vitro plaque reduction assay 13 Brazilian propolis ethanolic extracts were screened, four of which displayed an activity against influenza A/PR/8/34 (H1N1) virus. The effectiveness of the oral administration of the in vitro selected ethanolic extracts was further evaluated in DBA/2 CR mice infected with the same viral strain. Only one out of four candidate extracts showed a potential dose‐dependent anti‐influenza efficacy, without toxicity, in the murine model. At the dose of 10 mg/kg, propolis extract reduced virus titers in bronchoalveolar lavage fluid as effectively as oseltamivir 1 mg/kg. These doses are comparable to those used in humans as dietary supplement and therapeutic agent, respectively.^99^ This study, corroborated by both in vitro and in vivo evidence, is particularly significant since previous investigations had shown only negligible effects of other propolis types against influenza A/PR/8/34 (H1N1) virus. Unfortunately, no further evaluation to assess the peculiar components responsible for this effect has been reported. In another study, Urushisaki et al.^100^ investigated the efficacy of green Brazilian propolis aqueous extract against influenza A/WSN/33 (H1N1) virus. When tested in vitro in infected MDCK cells, propolis significantly increased cell survival. Then, to identify the specific components responsible for the activity, the authors screened individual isolated compounds. Among the major components of the extract, caffeoylquinic acids (including chlorogenic, 3,4‐dicaffeoylquinic, 3,5‐dicaffeoylquinic, 4,5‐dicaffeoylquinic and 3,4,5‐tricaffeoylquinic acids) are predominant, and 3,4‐dicaffeoylquinic acid, the most represented compound, was identified as the putative actor of the anti‐influenza effect. Since quinic acid was found to be ineffective, whereas caffeic acid displayed an anti‐influenza activity, the caffeoyl moiety might be indispensable in terms of effectiveness. Interestingly, the measurement of the relative amount of viral RNA did not show a significant reduction as a result of the treatment. For this reason, it may be speculated that propolis exerted no direct effect on virus particles, nor interacted with viral components, but rather enhanced cell resistivity via the activation/inactivation of the cellular process yet to be unraveled.^100^ The same research group further explored the ability of green Brazilian propolis and 3,4‐dicaffeoylquinic acid to act against influenza A virus, apparently without influencing the viral components. In vivo, aqueous and ethanolic extracts, and 3,4‐dicaffeoylquinic acid increased the survival times of infected BALB/c mice. In an attempt to explain the mechanism of action of 3,4‐dicaffeoylquinic acid, the mRNA expression in lungs of viral hemagglutinin was found to be moderately decreased, while the mRNA of TRAIL, a proapoptotic factor with viral clearance activity, was increased. Therefore, the authors confirmed the anti‐influenza activities of green Brazilian propolis extracts and hypothesized that their mode of action, at least in part, included two mechanisms: an unknown cytoprotective effect and the enhancement of viral clearance via TRAIL overexpression, both possibly induced by 3,4‐dicaffeoylquinic acid. Also, immunomodulatory and anti‐inflammatory effects might not be excluded. Notably, such a mechanism, based on the enhancement of self‐defense machineries of the host, might overcome the problems deriving from the emerging drug‐resistance of influenza viruses.^101^ Finally, Kai et al.^102^ assessed in vitro and in vivo the anti‐influenza efficacy of the ethanolic extract of Brazilian propolis from *M. euosma* (O. Berg) D. Legrand, and its components, against oseltamivir‐ and peramivir‐sensitive [A/PR/8/34 (H1N1) and A/Toyama/26/2011 (H1N1)], and oseltamivir and peramivir‐resistant [A/Toyama/129/2011 (H1N1)] influenza A virus. Apigenin, kaempferol, and *p‐*coumaric acid exhibited significant antiviral activity against all viral strains in a plaque reduction assay; however, kaempferol did not interfere with virus adsorption or invasion in vitro. The oral administration of kaempferol was significantly effective in prolonging survival times and reducing viral titers in bronchoalveolar lavage fluids of BALB/c mice infected with influenza A/PR/8/34 (H1N1) virus.^102^ Intriguingly, although not taken into consideration by the authors, this type of propolis is usually characterized by the presence of moronic acid, an antiviral triterpenoid with proven activity against HBV, HSV‐1, and HIV. Moreover, these findings reinforce the evidence that propolis of Brazilian origin appears to be more effective against H1N1 strains of influenza A virus.

#### 3.3.2 Pneumoviridae

Takeshita et al.^103^ examined in vitro and in vivo the effect of the dietary supplementation of Brazilian propolis extract against respiratory syncytial virus (RSV) infection. In vitro, propolis extract did not show anti‐RSV activity at non‐cytotoxic concentrations in an assay of plaque reduction in HEp‐2 cells. Nevertheless, in vivo, IFN‐γ, pro‐inflammatory cytokine (TNF‐α and IL‐6), and T_h_2 cytokine (IL‐4 and IL‐10) levels in bronchoalveolar lavage fluid of RSV infected BALB/c mice treated with propolis were lower than those in the control. The effect was particularly significant in the case of IFN‐γ, IL‐6, and IL‐10. Interestingly, propolis treatment did not affect the production of anti‐RSV antibodies.^103^ Altogether, these results support the hypothesis of an effect on the host immune system, rather than a direct antiviral one, and demonstrate that propolis administration may be equally beneficial in attenuating the inflammatory drawbacks of viral diseases.

### 3.4 Single‐stranded RNA retroviruses

#### 3.4.1 Retroviridae

Gekker et al.^104^ investigated the activity of ethanolic extracts from propolis of different geographical origin (Minnesota – USA; Rio Grande do Sul, Rio de Janeiro, Minas Gerais – Brazil; China) against human immunodeficiency virus type 1 (HIV‐1), the lentivirus responsible for AIDS. Minnesota propolis inhibited viral expression in a concentration‐dependent manner in CD4^+^ lymphocyte (85% suppression) and microglial (98% suppression) cell cultures at a concentration of 66.6 µg/ml. Similar anti‐HIV‐1 activity was observed with propolis samples from other geographical regions. The mechanism of propolis activity in CD4^+^ lymphocytes involved, at least in part, the inhibition of viral entry. Moreover, propolis displayed an additive effect with the reverse transcriptase inhibitor zidovudine but had no noticeable effect on the protease inhibitor indinavir.^104^ In the study by Harish *et al*., the authors showed the ability of propolis to suppress HIV‐1 replication in vitro in CEM cells. At high concentrations, propolis abolished syncytium formation, whereas at lower ones it was inhibited in a concentration‐dependent manner. Additionally, propolis decreased p24 antigen production by 90%–100% in a concentration‐dependent manner.^105^

In an in vitro screening of natural products against HIV‐1 in H9 T‐cell line, Ito et al.^106^ demonstrated the activity (EC_50_ <0.1 µg/ml) of a methanolic extract of propolis collected in southern Brazil, whose main botanical source was *M. euosma* (O. Berg) D. Legrand. The authors identified in the extract several triterpenoids among which moronic acid was found to be the major anti‐HIV substance.^106^ Notably, Bevirimat, a derivative of betulinic acid, another triterpenoid, is under development as anti‐HIV drug, showing the potential also of moronic acid. Silva et al.^107^ investigated in vitro the efficacy of extracts of Brazilian propolis from Ceará state, obtained with solvents of increasing polarity (hexane, chloroform, ethyl acetate, and methanol), in inhibiting HIV‐1 reverse transcriptase. The ethyl acetate‐soluble fraction exhibited the highest anti‐HIV activity and was further fractionated by column chromatography. Among isolated compounds isorhamnetin exhibited a moderate inhibitory effect against HIV‐1 reverse transcriptase (56.99 ± 3.91%), followed by naringenin (44.22 ± 1.71%), quercetin (43.41 ± 4.56%), and diprenylcinnamic acid (41.59 ± 2.59%).^107^ Díaz*‐*Carballo et al.^20^ analyzed in vitro the anti‐retroviral activity of two polyisoprenylated acylphloroglucinols, 7‐epi‐nemorosone, and plukenetione A, isolated from Caribbean propolis. The antiretroviral activity was studied on lentiviral particles produced in HEK 293T cells from a SIV‐based vector, while the antiviral activity was studied in CEMx174‐SEAP cells infected with the wild type HIV‐1 strain NL4‐3. Both 7‐epi‐nemorosone and plukenetione A were found to be potent anti‐lentiviral agents. However, although the two compounds share the same adamantane moiety, only plukenetione A could effectively inhibit the reverse transcriptase.^20^ The significance of these in vitro findings should be confirmed with in vivo studies aimed at assessing the real exploitability of the observed molecular mechanisms in clinical practice. Nevertheless, the anti‐HIV activity of unusual or low occurring propolis components might arouse interest for the discovery of innovative lead compounds.

## 4 IMMUNOMODULATORY ACTIVITY

The results of in vivo and clinical studies on the antiviral activity of propolis, presented in the previous section, have often highlighted other mechanisms of action involving the modulation of the host immune responses, an effect which complements direct antiviral activities but sometimes appears to be independent and even more relevant. Some of the earliest evidence of propolis immunomodulatory activities was obtained by the group of Popov in the early 1990s. In a series of studies, the authors examined in vitro and in vivo the immunomodulatory action of a water‐soluble propolis derivative on complement activity. In vitro, the extract inhibited classical and alternative complement pathways in a concentration‐dependent manner. In particular, the inhibition of the classical pathway was stronger. Moreover, the extract diminished C3 protein functional activity.^108^ In vivo, the extract was administered intravenously, intraperitoneally, and orally to ICR mice. An alteration of serum alternative pathway complement levels was observed. Interestingly, a significant reduction of acute inflammation in zymosan‐induced paw edema was obtained after oral administration, a condition in which serum complement levels were not influenced, demonstrating that the effect was strongly dependent on the route of application.^109^ Although partial, these results paved the way to the comprehension of propolis effects on the host immune response.

In the late 1990s Brätter et al.^110^ conducted the first clinical open trial to find evidence for the prophylactic immunostimulant efficacy of propolis oral administration. Although the cytokine plasma levels did not significantly change during the study, propolis led to a significant increase of both the spontaneous (TNF‐α, IL‐6, and IL‐8) and LPS‐induced (TNF‐α, IL‐6, IL‐8, and IL‐1β) cytokine secretion capacity following short‐term ex vivo culture of peripheral blood leukocytes, demonstrating that propolis led to a time‐dependent enhanced immune reactivity without undesired side effects.^110^ The most recent updates consist of two systematic reviews in which the effect of propolis supplementation on C reactive protein, TNF‐α, IL‐1, and IL‐6 levels were investigated. The meta‐analyses of randomized clinical trials highlighted a significant reduction in IL‐6, C reactive protein, and TNF‐α serum levels following propolis administration, whereas propolis did not exert any significant effect on IL‐1.^111^, ^112^

The vast majority of evidence in this context, though, is still anchored to the preclinical level. Consequently, in the following paragraphs in vitro, ex vivo, and in vivo findings are presented, grouped according to the propolis origin and type, to highlight any peculiarity of each. This subdivision has been adopted due to the fact that propolis is a poorly standardized natural product, characterized by extreme variability and multifariousness of composition, that cannot be reduced to the mere sum of components active on specific molecular targets. Despite the similarities in composition, the overall effect is, often and however, dramatically different.

What emerges from this section is that most of the evidence is related to Brazilian propolis; however, among the thirteen known subtypes, in practice only the red and, especially, the green ones have been investigated in this context, at the expense of other varieties still poorly characterized.

**Figure 2:**
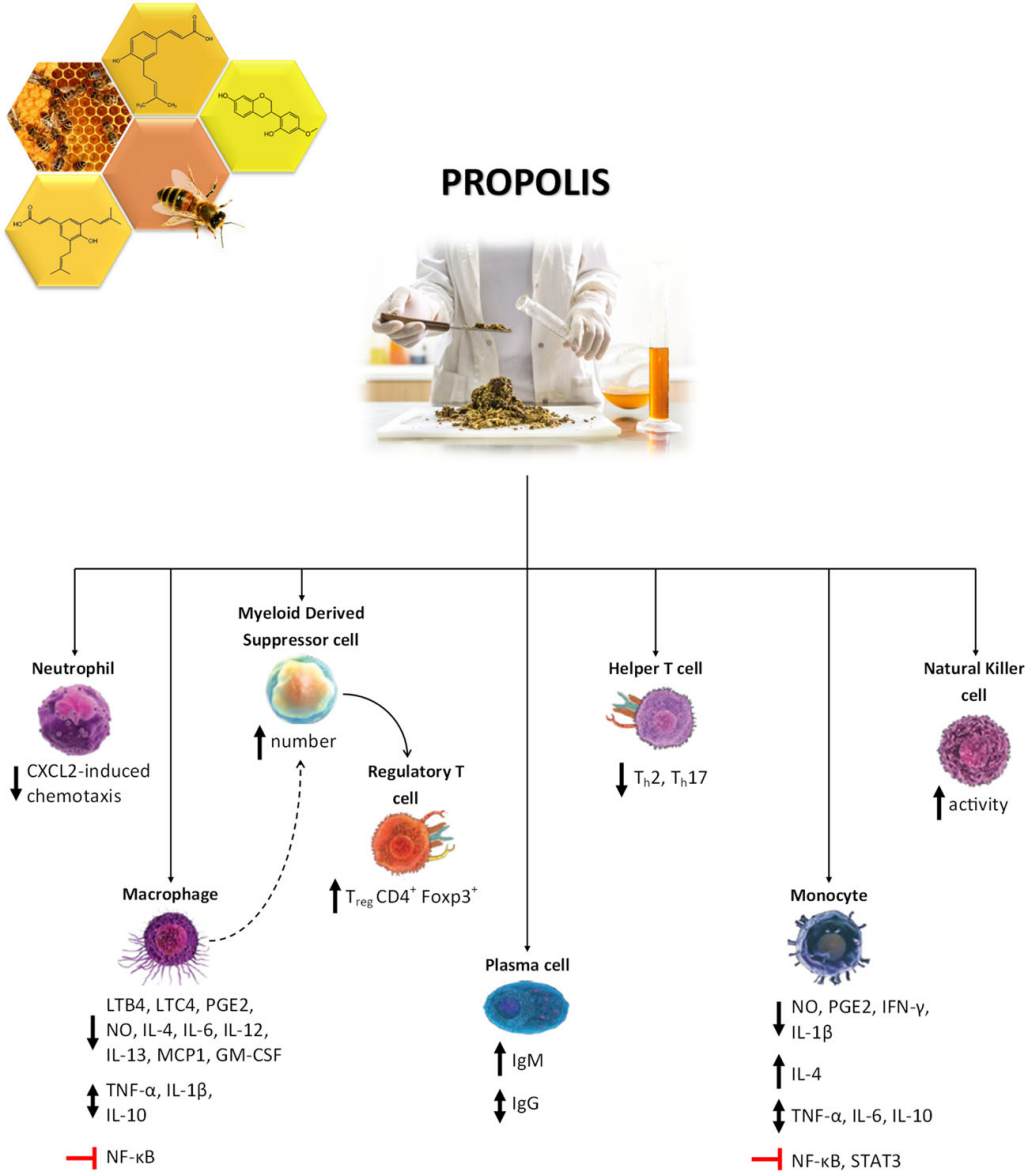
An overview of the immunomodulatory effects of propolis hydroalcoholic/ethanolic extracts (based on in vivo and ex vivo evidence) [Color figure can be viewed at wileyonlinelibrary.com]

The principal modes of action that emerged from preclinical studies are collectively summarized in Figure 2.

### 4.1 Brazilian propolis (São Paulo State)

A considerable number of studies have been conducted on Brazilian propolis collected in the UNESP (Universitate Estadual Paulista) campus in Botucatu, São Paulo State. Its main components include hydroxycinnamic acids (caffeic, ferulic, and *p*‐coumaric acids) and caffeoylquinic acids (1,3‐dicaffeoylquinic acid, 4,5‐dicaffeoylquinic acid, and 3,4,5‐tricaffeoylquinic acid), whereas only trace amounts of flavonoids were detected. Even though not openly stated by the authors, the geographical provenance and the chemical composition suggest the classification of this propolis within subtype 12 of Brazilian propolis, that is green propolis mainly derived from *Baccharis dracunculifolia* DC.

Propolis hydroalcoholic (70% ethanol) extract exerted in vitro a direct inhibitory effect on BALB/c mouse splenocyte proliferation, also in presence of concanavalin A, a mitogenic inductor able to enhance IFN‐γ, IL‐2, IL‐4, IL‐6, and TNF‐α production. In ex vivo experiments, when mice were treated intraperitoneally for three days with propolis hydroalcoholic solution, inhibition of concanavalin A‐induced splenocyte proliferation could be only seen at lower doses (2.5–5 mg/kg). Propolis alone did not induce IFN‐γ release in cell culture supernatants, but concanavalin A‐stimulated splenocytes from propolis‐treated mice produced significantly more IFN‐γ than the stimulated controls.^113^ TLR‐2 and TLR‐4 expression, and IL‐1β and IL‐6 (only in splenocytes) production were upregulated in splenocytes and peritoneal macrophages, possibly indicating that propolis modulated the mechanisms of the innate immunity, activating the initial steps of the immune response.^114^ In unstimulated splenic tissue, propolis did not affect T_h_1 (IL‐2 and IFN‐γ) and T_h_2 (IL‐4 and IL‐10) mRNA expression; IFN‐γ mRNA could not be even detected in experimental groups. However, the analysis of cytokine production in ex vivo cultured splenocytes showed a reduction in IFN‐γ basal production, whereas no changes were detected in IL‐2, IL‐4, and IL‐10.^115^ Pagliarone et al.^116^ evaluated the same parameters in splenic cells of acutely stressed BALB/c mice treated per os with propolis extract for 3 days after being subjected to a restraint stress protocol. Higher corticosterone concentrations were characteristic markers of the stressed group, treated or not with propolis, in comparison with the control group. As far as it concerns T_h_1 cytokine production, no alterations were observed regarding IL‐2, while IFN‐γ production was inhibited in stressed mice even after propolis treatment. As to T_h_2 cytokines, IL‐4 production was inhibited in stressed mice, but levels were normalized by propolis treatment. No significant differences were found in IL‐10 production. These results suggest the inability of propolis extract counteract the immunosuppressive effect on IFN‐γ production in acutely stressed mice. Nevertheless, propolis could increase IL‐4 production, thus favoring the humoral immune response under stress.^116^ The potential effect of a preventive treatment was not considered, since propolis was administered only following the stressful event. On the other hand, Missima and Sforcin considered the immunomodulatory effects on macrophages and lymphoid organs in chronically stressed BALB/c mice subjected to immobilization stress. Stressed mice showed greater H_2_O_2_ production by peritoneal macrophages. Propolis treatment further increased H_2_O_2_ generation but inhibited NO production by these cells. Histopathological analysis showed no alterations in thymus, bone marrow, and adrenal glands, but propolis treatment counteracted the alterations found in the spleen, as splenic germinal centers appeared to be increased.^117^ In chronically stressed C57BL/6 mice, propolis administration prevented the downregulation of splenic TLR‐2 and TLR‐4 mRNA expression, favoring the initial steps of the immune response, thus reinforcing the evidence obtained in in vitro and ex vivo experiments.^118^ Bachiega et al.^119^ investigated in vitro the effects of the extract and two isolated compounds (cinnamic and *p*‐coumaric acids) on cytokine production (IL‐1β, IL‐6, and IL‐10) by peritoneal macrophages from BALB/c mice, with or without LPS challenge. Propolis, cinnamic acid, and *p*‐coumaric acid increased IL‐1β production by macrophages, suggesting an immunostimulatory action. Cinnamic acid and *p*‐coumaric acid alone induced a higher IL‐1β production compared with propolis, with the maximum effect at the lowest concentration tested. Conversely, propolis and its constituents inhibited IL‐6 secretion by peritoneal macrophages in a concentration‐dependent manner, in basal conditions and in both pre‐ and post‐treatments upon LPS challenge. IL‐10 production was significantly inhibited by propolis and *p*‐coumaric acid in basal conditions. Upon LPS challenge, propolis inhibited IL‐10 production in pre‐ and post‐treatments, whereas cinnamic and *p*‐coumaric acids were effective only in post‐treatments. Since high levels of IL‐10 are characteristic of immunosuppressed patients, the inhibitory action of propolis on IL‐10 production may be useful to prevent infections. These data showed that propolis may exert anti‐inflammatory and immunomodulatory effects, mainly thanks to the synergy existing among its components, in particular cinnamic and *p*‐coumaric acids, which are probably involved in the effect on cytokine production.^119^

A consistent number of studies considered the immunomodulatory effect of propolis hydroalcoholic extract on human peripheral blood mononuclear cells (PBMCs). The treatment with propolis at low concentration (5 µg/ml) reduced NO production in PBMCs from healthy donors, while at the highest concentration tested (25 µg/ml) increased IL‐4 and abolished, though not significantly, IL‐10 production. Levels of IL‐2, TNF‐α, IFN‐γ, and IL‐17 were unaffected.^120^ However, Amarante et al.^121^ found that IFN‐γ mRNA expression in presence of propolis extract was markedly downregulated. Búfalo et al.^122^ further explored the effect on the initial events of the immune response, evaluating receptor expression (TLR‐2/4, HLA‐DR, and CD80) and cytokine production (TNF‐α and IL‐10) in human PBMCs. Propolis had no effect on TLR‐2 and HLA‐DR expression, whereas upregulated the percentage of TLR‐4 and CD80. The treatment significantly stimulated TNF‐α production at lower concentrations (5–50 μg/ml), while a significant inhibitory activity emerged at 100 μg/ml. IL‐10 production was stimulated at 5–10 μg/ml of propolis extract and inhibited at 50–100 μg/ml. Additionally, the authors demonstrated that cytokine production was dependent on TLR‐4 and could be abolished by blocking TLR‐4.^122^ Conte et al.,^123^ after having identified *Araucaria angustifolia* (Bertol.) Kuntze, *Baccharis dracunculifolia* D.C., and *Corymbia citriodora* (Hook.) K.D. Hill and L.A.S. Johnson as the main botanical sources of the propolis used in the study, evaluated the immunomodulatory effects of the hydroalcoholic (70% ethanol) extract on monocytes treated to induce different T helper profiles (T_h_1, T_h_2, T_h_17, and T_reg_). Propolis alone did not alter the basal expression of TLR‐2, TLR‐4, HLA‐DR, CD40, and CD80. When monocytes were treated with retinoic acid (RA) to induce a T_reg_ profile, propolis maintained the suppressive function of RA on TLR‐2 expression and TNF‐α production, whereas exerted conflicting effects on CD80 depending on propolis concentration. Propolis inhibited RA‐stimulated IL‐6 production in a dose‐dependent manner, suggesting a possible strategy to control inflammatory conditions. In monocytes treated with the B subunit of *Escherichia coli* heat‐labile enterotoxin (EtxB) to induce a T_h_2 profile, propolis suppressed TNF‐α production, demonstrating once again that it may favor an anti‐inflammatory milieu. In LPS‐treated T_h_17 polarized cells, propolis decreased CD40 expression and sustained LPS‐induced IL‐6 production. Nonetheless, other authors have demonstrated an anti‐inflammatory effect of propolis in response to LPS. Finally, when monocytes were exposed to melanoma‐associated antigen‐1 (MAGE‐1) to induce a T_h_1 profile, propolis decreased CD40 expression, inhibited TNF‐α and IL‐6 release, and increased IL‐10 production, thus favoring a deactivating status important to avoid damage to the host. The immunomodulatory activity of propolis was shown to be independent from autophagy, since LC3 expression was not induced. In addition, propolis inhibited NF‐κB expression, downregulating monocyte inflammatory activity.^123^ Even though the signaling pathways modulated in differentially polarized monocytes were not fully elucidated, propolis exerted an immunomodulatory activity oriented toward an anti‐inflammatory profile. These results, which appear sometimes inconsistent, show that the effects of propolis extract may be different, and even opposite, in relation to treatment concentrations and other less predictable experimental variables.

Among isolated constituents of propolis, caffeic acid downregulated TLR‐2 and HLA‐DR expression without exerting any effect on TLR‐4 and CD80 expression. The treatment inhibited TNF‐α and IL‐10 production at all concentrations. In addition, while the first was affected by TLR‐4, but not TLR‐2 blockage, the latter was altered neither by TLR‐2 nor TLR‐4 blockage.^124^ Cinnamic acid downregulated TLR‐2 expression; on the other hand, TLR‐4 expression was increased. HLA‐DR expression was inhibited at all tested concentrations, while CD80 was only at the highest one (100 µg/ml). Both TNF‐α and IL‐10 production were inhibited by cinnamic acid treatment, but this effect was abolished by TLR‐4 blockage, thus suggesting, at least in part, its involvement in cinnamic acid activity on human monocytes.^125^ Altogether these results demonstrate that propolis isolated compounds may exert immunomodulatory activities on PBMCs, but nonetheless, most of the synergistic effects are still unknown, as well as the scale and significance of these effects in vivo and at a clinical level. Cardoso et al.^126^ investigated the involvement of various phenolic acids (caffeic, dihydrocinnamic, and *p*‐coumaric acids), alone or in combination, in the activity of propolis on resting and LPS‐activated human PMBCs. Caffeic acid combinations enhanced TNF‐α production by resting PBMCs, but not by LPS‐stimulated cells. All treatments upregulated IL‐10 in resting monocytes, in particular caffeic acid combinations exerted an effect actually higher than the one induced by propolis extract, whereas neither propolis nor phenolic acids affected IL‐10 production by LPS‐stimulated monocytes. Propolis, dihydrocinnamic acid, *p*‐coumaric acid, and a combination of caffeic and dihydrocinnamic acids significantly stimulated IL‐6 production by resting monocytes. However, in LPS‐stimulated cells, the treatments only slightly downregulated IL‐6. Propolis and phenolic acids did not influence the expression of HLA‐DR, CD80 and TLR‐2; however, the combination of caffeic and dihydrocinnamic acids upregulated LPS‐induced TLR‐4 expression. These results reaffirm the ability of Brazilian propolis extract to activate human monocytes and demonstrate that phenolic acids are differentially involved in its activity.^126^

Finally, Conti et al.^127^ compared the immunomodulatory effects of the hydroalcoholic (70% ethanol) extract of Brazilian propolis collected in Botucatu with methanolic extracts of Mexican and red Cuban propolis on cytokine production in human monocytes. Brazilian propolis stimulated TNF‐α production starting from a concentration of 10 μg/ml, Cuban propolis exerted a stimulatory effect at all concentrations, whereas the treatment with Mexican propolis inhibited TNF‐α production, significantly suppressing the basal levels in comparison with control cells. Brazilian and Mexican propolis stimulated IL‐10 production in a concentration‐dependent manner, whereas Cuban propolis showed inhibitory activity. In general, propolis immunomodulatory actions depend on its origin and consequently chemical composition, and although these findings seem to be contradictory, differences in cytokine production may be due to different compositions and synergistic or antagonistic effects among the components. Nonetheless, Brazilian, Cuban, and Mexican propolis contain different constituents that may exert mild pro‐inflammatory effects, useful to stimulate the initial events of the immune response or anti‐inflammatory activity, depending on their concentration.^127^

Such considerations on propolis range of effects may well apply also to the particular case of Brazilian propolis collected in Botucatu and used in the studies presented in this section. Despite the same provenance, the variance among the responses obtained in murine or human, resting or variously activated, immune cells might be ascribed on the one hand to differences inherent in experimental models, on the other to the summation of different activities exerted by individual components and synergisms still to be unraveled. An exemplary case is that of the effect on IL‐10 levels, found to be unchanged, elevated, or reduced, each time with plausible scientific explanations. However, what seems certain is the ability of this propolis type to modulate the mechanisms of the innate immunity, possibly activating the initial steps of the immune response, and that these immunomodulatory and anti‐inflammatory effects are mainly the result of additive, synergistic and antagonistic interactions among individual components, which define the overall pharmacological activity. Nonetheless, more studies, both preclinical and especially clinical, are needed to definitively elucidate the direction and entity of the effects and their therapeutical exploitability.

### 4.2 Brazilian propolis (other origins)

Origin, subtype, and chemical composition of propolis are crucial factors to understand the relevance of the reported findings and direct the overall interpretation of propolis biological effects and therapeutic utility. However, the indication of such information is often lacking or incomplete. In the present section, evidence has been tentatively organized by inferring missing information, trying to combine studies considering similar propolis extracts.

In a study by Takagi et al.,^128^ Brazilian propolis hydroalcoholic (70% ethanol) extract suppressed IgG production in C3H/HeNCrj mice, while the amount of IgM was found to be significantly higher in comparison with the control group. In C57BL/6CrSlc mice treated with propolis, the number of CD4^+^ cells increased, both in basal condition and after total‐body X‐ray irradiation (2 Gy), while the number of CD8^+^ cells increased dramatically in basal condition, but only slightly after irradiation, without reaching the levels of nonirradiated controls. According to the authors, propolis could activate macrophages and stimulate IFN‐γ production, thus resulting in a decrease in IgG and IgM production.^128^ The dietary supplementation of raw green propolis improved the innate and adaptative immunity in aged Kunming mice. After 4 weeks of treatment, propolis administration promoted phagocytosis in peritoneal macrophages and significantly increased phagocytic indexes in comparison with the control. Splenic T‐cell proliferation was only slightly augmented, while splenic NK cells were unaffected. Serum IgG was dramatically increased in mice receiving propolis, whereas no significant change was detected in IgM. Serum hemolysins were found to be higher in treated mice, while IL‐1β, IFN‐γ, and IL‐4 levels were unaffected. These results indicated that green Brazilian propolis was effective in improving innate and adaptive immunity in aged mice, especially at a dose of 157.4 mg/kg.^129^ These two studies present contrasting evidence, especially in the case of a preferential effect on IgG or IgM and the influence on IFN‐γ production, probably owing to inherent differences in animal models. Nevertheless, what is particularly noticeable is the ability of Brazilian propolis to exert an immunomodulatory effect, which is still to be completely unraveled.

In vitro, green Brazilian propolis extract significantly reduced the number of CD4^+^ IL‐17^+^ T‐cells in BALB/c mouse isolated splenocytes subjected to T_h_17‐polarizing conditions. In addition, propolis directly inhibited IL‐6‐induced STAT3 phosphorylation in CD4^+^ T‐cells, a pathway critical for T_h_17 differentiation.^130^ Since T_h_17 cells are a subset of pro‐inflammatory T helper cells, the hinderance of their differentiation constitutes an intriguing mechanism possibly involved in propolis anti‐inflammatory activity on immune cells. Intraperitoneal administration of green Brazilian propolis ethanolic extract induced CD11b^+^ Gr‐1^+^ myeloid‐derived suppressor cells in the visceral adipose tissue and in the peritoneal cavity of C57BL/6 mice. In vitro, propolis stimulated also the J774A.1 M1 macrophage‐like cell line differentiation into myeloid‐derived suppressor cells. Among several isolated compounds, this effect could be specifically attributed to kaempferol and was further confirmed in vivo. The intraperitoneal injection of kaempferol, in fact, was able to elicit accumulation of myeloid‐derived suppressor cells in the visceral adipose tissue.^131^ Myeloid‐derived suppressor cells are known to inhibit T cell activation and proliferation by infiltrating inflammatory sites, thus potentially limiting the inflammation induced by an immune over‐responsiveness in case of viral and bacterial infections. An ethanolic extract of green Brazilian propolis collected in Minas Gerais State significantly inhibited paw and ear edema induced by various stimuli in Swiss mice after both intraperitoneal and oral administration. Moreover, propolis decreased the number of peritoneal neutrophils in carrageenan‐induced peritonitis, an effect confirmed by reduced myeloperoxidase activity. In vitro, treatment with propolis decreased NO generation by LPS‐activated RAW 264.7 macrophages and suppressed NF‐κB‐driven transcription in TNF‐α‐stimulated HEK 293 cells.^132^ In search of molecules responsible for such anti‐inflammatory effect, intraperitoneal administration of artepillin C, one of the most abundant compounds found in green Brazilian propolis, significantly reduced carrageenan‐induced paw edema in Swiss mice and decreased neutrophil recruitment in carrageenan‐induced peritonitis, proportionally to the decrease in myeloperoxidase activity. Moreover, PGE2 secretion in the peritoneal exudate was significantly inhibited. In vitro, artepillin C impaired NO generation by LPS‐activated RAW 264.7 macrophages and NF‐κB transcriptional activity in TNF‐α‐stimulated HEK 293 cells. Notably, the bioavailability of a single oral dose of artepillin C was measured in mice, and it was found that the absorption was sufficient to produce biological effects,^28^ thus providing evidence for the attribution of the activity reported in vivo to this molecule. Adachi et al.^133^ generated a conditional Ca^2+^ biosensor transgenic mouse line (CD11c‐Cre/YC3.60^flox^) to assess in vitro and in vivo the immunological effects of green Brazilian propolis ethanolic extract (standardized to contain a minimum of 8.0% artepillin C). In vitro, propolis‐induced Ca^2+^ signaling in B cells and dendritic cells. The intravital imaging of Peyer's patches showed that both the intraperitoneal injection and the oral administration of propolis augmented Ca^2+^ signaling in CD11c^+^ cells, suggesting an immune‐stimulating activity. The effect might be due to the activation of transient receptor potential ankyrin 1 (TRPA1) channels, which induce Ca^2+^ flux, by artepillin C. Despite propolis‐induced Ca^2+^ signaling in immune cells, it did not induce the expression of activation markers on lymphocytes and their proliferation but rather triggered dendritic cells, which may activate T‐cells and/or B cells via their cognate interactions. Thus, propolis may stimulate the immune responses both directly and indirectly.^133^ Finally, Mikami et al.^134^ investigated the immunomodulatory activity of green Brazilian propolis ethanolic extract (standardized to contain 8.0% artepillin C and 0.14% culifolin) on Foxp3^+^ regulatory T cells (T_reg_). Propolis did not affect Foxp3 expression in induced regulatory T cells (iT_reg_) and natural regulatory T cells (nT_reg_), but rather supported T_reg_ expansion and activation through the upregulation of TNFR2 expression via IRF4/cMyc pathway, with artepillin C being a major actor of this effect. TNRF2 has in fact been recently reported as a T_reg_‐specific receptor that contributes to the anti‐inflammatory immunosuppressive feedback exerted by T_reg_ cells.^134^ Once again, it was demonstrated that the same propolis type, that is, artepillin C‐rich green Brazilian propolis, may exert different but equally interesting effects on different immune cells subtypes, on the basis of the specific context taken under consideration.

In turn, Bueno‐Silva et al.^36^ investigated in vivo the main pathways of action of red Brazilian propolis hydroalcoholic (80% ethanol) extract on the modulation of neutrophil migration into the peritoneal cavity of BALB/c mice. Propolis extract, administered subcutaneously before intraperitoneal carrageenan injection, prevented neutrophil migration into the peritoneal cavity, significantly reduced leukocyte rolling and adhesion in the mesenteric microcirculation, and inhibited the release of TNF‐α, IL‐1β, CXCL1, and CXCL2. In vitro, propolis reduced CXCL2‐induced chemotaxis of bone marrow‐isolated neutrophils, without affecting cell viability.^36^ This anti‐inflammatory effect is in many respects analogous to the one previously described for green propolis. The same group also studied the effect on the activity of peritoneal macrophage isolated from C57BL/6 mice and activated with LPS. Propolis treatment reduced NO production by 65%, without aﬀecting cell viability, and decreased the production of IL1‐β, IL‐4, IL‐6, IL‐12, IL1‐3, MCP1, and GM‐CSF.^135^ One of the principal constituents of red Brazilian propolis, neovestitol, was shown to specifically exert immune‐modulatory effects on LPS‐activated RAW 264.7 murine macrophages in vitro, reducing NO production by 60% without aﬀecting cell viability and decreasing GM‐CSF, IFN‐γ, IL1‐β, IL‐4, TNF‐α, and IL‐6 levels, while increasing IL‐10 production,^136^ configuring a pro‐resolving mechanism of action. In a similar study, also vestitol inhibited NO production by 83%, without affecting cell viability, and reduced GM‐CSF, IL‐6, TNF‐α, IL‐4, and TGF‐β levels, whereas increased IL‐10 release. Moreover, vestitol modulated the expression of several genes related to NF‐κB pathway, NO synthase, and the inhibition of leukocyte transmigration, diminished the activation of NF‐κB and Erk 1/2 pathways, and induced macrophages into M2‐like polarization.^137^

Not only hydroalcoholic and ethanolic extracts of Brazilian propolis have been considered in experimental studies, but also aqueous extracts and water‐soluble derivatives. Machado et al.^138^ examined the immunomodulatory and anti‐inflammatory activities of different green Brazilian propolis aqueous extracts administrated per os. Cotton pellet granuloma in Swiss mice and LPS‐induced pulmonary inflammation in BALB/c mice were chosen as models of chronic and acute inflammation. One of the extracts, obtained through alkaline hydrolysis and rich in *p*‐coumaric acid, demonstrated anti‐inflammatory properties in both models. In the cotton pellet granuloma model, the extract reduced the total/dry weight of granuloma and edema severity, increased leukocytes in circulating blood and lymphoid organs, and reduced platelet counts. In LPS‐induced pulmonary inflammation, the treatment with propolis extract decreased macrophages, neutrophils, and lymphocytes in bronchoalveolar lavage fluid. Furthermore, the treatment induced a reduction in TNF‐α and IL‐6 levels, and increased TGF‐β and IL‐10.^138^ Takeda et al.^139^ assayed in vivo the ability of a water‐soluble derivative of propolis to increase the cytotoxic activity of natural killer (NK) cells, which have a chief role in immune surveillance against viral infections. In addition, the authors assessed the effects of the major components of the water‐soluble propolis derivative (artepillin C, drupanin, and *p*‐coumaric acid). The cytotoxic activity of NK cells was increased following propolis oral administration in BALB/c wild type mice, but not in IFN‐γ^−/−^ or IFN‐γR^−/−^ mice, suggesting a critical role for IFN‐γ in the augmentation of NK cell cytotoxicity. However, the proliferation of NK cells was unaffected. The treatment with artepillin C or *p*‐coumaric acid, but not drupanin, augmented NK cell cytotoxicity in a manner similar to propolis extract and to the mixture of the three isolated components.^139^ In the last two cases, although not explicitly mentioned, the extract can be matched to the green propolis subtype, owing to the reported chemical composition.

### 4.3 Chinese propolis

The composition of Chinese propolis is generally similar to that of the European poplar type, rich in flavonoids and phenolic acids esters, and glycerides. An extract of Chinese propolis, characterized by the presence of galangin, chrysin, and pinocembrin, was able to inhibit the secretion of TNF‐α, IL‐1β, and chiefly IL‐6, from LPS‐stimulated human PBMCs, whereas it had no effect on IL‐8. Additionally, propolis significantly reduced IL‐10, PGE2, and NO levels and completely abrogated IFN‐γ production.^140^

Shi et al.^141^ evaluated the properties of 15 different methanolic extracts of Chinese propolis from various regions. All samples signiﬁcantly diﬀered in their total phenolic and total ﬂavonoid contents, as well as in their phytochemical proﬁles. However, all the propolis extracts showed to a greater or lesser extent a significant inhibitory effect on IL‐1β, IL‐6, and COX‐2 mRNA expression in LPS‐stimulated RAW 264.7 murine macrophages.^141^ The same research group investigated in the same model the anti‐inflammatory properties of five novel glycerol esters (2‐acetyl‐1‐coumaroyl‐3‐cinnamoylglycerol, (+)−2‐acetyl‐1‐feruloyl‐3‐cinnamoylglycerol, (−)−2‐acetyl‐1‐feruloyl‐3‐cinnamoylglycerol, 2‐acetyl‐1,3‐dicinnamoylglycerol, and (−)−2acetyl‐1‐(E)‐feruloyl‐3‐(3″(ζ),16″)‐dihydroxy‐palmitoylglycerol) isolated from the methanolic extract of propolis collected in Wuhan. Both propolis extract and the isolated compounds showed a concentration‐dependent inhibitory effect on IL‐1β, IL‐6, and COX‐2 mRNA expression.^142^

Finally, Wang et al.^143^ investigated the activity of an ethanolic extract of poplar propolis collected in Shāndōng Province, which proved able to decrease IL‐6, IL‐10, iNOS, COX‐2, and MCP1 mRNA expression in LPS/IFN‐γ co‐stimulated RAW 264.7 murine macrophages. Additionally, lower doses of extract (5 μg/ml) slightly inhibited TNF‐α and G‐CSF mRNA expression, while higher doses (10 μg/ml) caused their dramatic increase. As to protein production, propolis significantly abolished IL‐6 and MCP1 levels; lower doses enhanced IL‐10 synthesis, whereas higher doses impaired it. Propolis pretreatment also significantly inhibited NO production and NF‐κB activation in TNF‐α‐stimulated HEK 293T cells and IL‐1β‐stimulated 293‐C6 cells. In vivo, the oral administration provided significant protective effects by attenuating lung histopathological changes and suppressing the secretion of LPS‐stimulated inflammatory cytokines, including IL‐6, IL‐10, MCP1, TNF‐α, and IL‐12 in ICR mice with endotoxemia.^143^ The results of these studies, all preclinical, have mainly highlighted the anti‐inflammatory properties of Chinese propolis, able to decrease both cytokine release and mRNA levels of pro‐inflammatory mediators in activated immune cells. Once again, the effects proved to be potentially different, and even opposite, in relation to treatment doses. Intriguingly, in vivo findings suggest the ability of this propolis type to possibly attenuate lung damage in case of severe inflammatory conditions involving systemic massive cytokine release. However, clinical trials should be undertaken to assess the actual therapeutical benefits.

### 4.4 Other propolis

In this section, the evidence related to propolis types of various origins, the limited number of which does not permit an individual separate discussion, is presented.

Iranian propolis ethanolic extract could inhibit the release of T_h_2 cytokines, IL‐13, and IL‐17 induced by *Aspergillus fumigatus* conidia in murine lung epithelial cells (TC‐1).^144^ Moreover, when the extract was administrated per os to tumor‐bearing BALB/c mice with disseminated *Candida albicans* infection, a condition in which the mean tumor size was significantly higher when compared with control group, propolis treatment significantly reduced tumor size in both *C. albicans*‐infected and noninfected mice. Propolis determined a significant decline in T_h_2 cytokines (IL‐4 and IL‐10) and a slight decrease in IL‐17 production, whereas a significant increment in TNF‐α and IFN‐γ levels was observed in comparison with control groups.^145^ The reduction of IL‐17 production may correlate with the inhibition of T_h_17 differentiation, an effect previously reported for Brazilian propolis. T_h_17 cells play a role in adaptive immunity, fungal infections, and promotion or regression of tumors in murine models, however, as mentioned earlier, might also be responsible for detrimental inflammatory effects.

Turkish propolis ethanolic extracts decreased neopterin release and tryptophan degradation mediated by indoleamine 2,3‐dioxygenase activity in PBMCs, both resting and treated with mitogenic phytohemagglutinin, and reduced TNF‐α and IFN‐γ production in stimulated cells. Indoleamine 2,3‐dioxygenase is a heme‐containing enzyme important for the defense against various pathogens, produced in response to inflammation, and able to inhibit T‐cell function and induce immune tolerance. Thus, the inhibition of its activity represents an immunostimulatory mechanism.^146^ A dimethyilsulfoxide extract of Turkish propolis reduced phorbol myristate acetate‐induced respiratory burst, and the consequent secretion of elastase, a neutrophilic marker of acute inflammatory responses, in isolated human polymorphonuclear leukocytes.^147^

Medjeber et al.^148^ assessed the effect of Algerian propolis hydroalcoholic (85% ethanol) extract in PBMCs from patients with celiac disease. Compared with control, PBMCs from patients showed higher NO and IFN‐γ levels, which were significantly decreased by propolis treatment. In addition, propolis extract significantly increased IL‐10 production, downregulated iNOS mRNA expression, and impaired NF‐κB and pSTAT‐3 transcription factors activity.^148^ In a model of carrageenan‐induced inflammation in Wistar rats, the oral pretreatment with the ethyl acetate fraction of an ethanolic extract of Algerian propolis from Tigzirt significantly reduced paw edema, counteracted white blood cell increment, and increased the enzymatic activity of superoxide dismutase, catalase, and glutathione peroxidase. Moreover, propolis reduced the levels of PGE‐2 and TNF‐α and the activity of myeloperoxidase in the peritoneal exudate. In particular, the authors demonstrated that propolis is able to both reduce the release of myeloperoxidase from neutrophils and directly inhibit its activity.^149^

The immunomodulatory properties of Moroccan propolis, mainly characterized by pinocembrin, chrysin, quercetin, and galangin, were evaluated in vitro by Touzani et al.^150^ Propolis extract alone showed no effect on the production of TNF‐α and IL‐6, as well as IL‐10, in human PBMCs. However, when PBMCs were stimulated with LPS, propolis significantly inhibited the secretion of TNF‐α and IL‐6, which at a concentration of 250 μg/mL declined toward basal levels, whereas IL‐10 production was strongly enhanced in a concentration‐dependent manner, thus indicating a selective anti‐inflammatory and pro‐resolving effect on activated immune cells.^150^

Shvarzbeyn and Huleihel^151^ evaluated in vitro the effect of an aqueous Israeli propolis extract and CAPE on NF‐κB induction by the human T‐cell lymphotropic virus type 1 (HTLV‐1), an oncogenic deltaretrovirus responsible for adult T‐cell leukemia and lymphoma. The viral protein Tax is a key factor in HTLV‐1 pathogenicity, and its oncogenic potential depends on the capacity to constitutively induce the transcriptional activity of NF‐κB factors. The results showed that both propolis extract and CAPE significantly inhibited the activation of NF‐κB‐dependent promoters induced by Tax in Jurkat and MT2 T‐cells. However, only propolis could efficiently inhibit also the Tax‐induced activation of SRF‐ and CREB‐dependent promoters. Moreover, propolis extract and CAPE strongly prevented Tax binding to IκBα and its consequent degradation. Nevertheless, neither propolis nor CAPE could interfere with the nuclear translocation of Tax or NF‐κB.^151^

Blonska et al.^152^ studied in vitro the effect of Polish propolis ethanolic extract and isolated ﬂavonoids (chrysin, galangin, kaempferol, and quercetin) on IL‐1β and iNOS gene expression in LPS‐activated J774.A1 murine macrophages. The treatments signiﬁcantly suppressed both IL‐1β and iNOS mRNA expression when compared to activated control macrophages. In addition, propolis significantly decreased IL‐1β protein synthesis and secretion in a concentration‐dependent manner. Among isolated derivatives, chrysin, kaempferol, and quercetin suppressed cytokine release, whereas galangin produced only a slight decrease. The most potent inhibitor of IL‐1β synthesis and NO generation was chrysin, which, together with the other tested ﬂavonoids, contribute to the anti‐inﬂammatory activity of propolis extract. NO generation was inhibited in a concentration‐dependent manner by both propolis and flavonoids.^152^

Draganova‐Filipova et al.^153^ evaluated Bulgarian propolis ethanolic extract immunomodulatory activity on PBMCs, assessing the percentage of T helper/inducer (CD4^+^ CD3^+^), T cytotoxic (CD8^+^ CD3^+^), B (CD19^+^ CD3^−^), and NK (CD56^+^ CD16^+^ CD3^−^) lymphocyte subsets, as well as the proportion of apoptotic (annexin V^+^) cells within each subset. Low concentrations of propolis (1–2.5 µg/ml) had a protective effect on the activity and proliferation of B cells but did not affect the percentage of CD4^+^ and CD8^+^ T‐cells. No negative effects could be seen on the proliferation and vitality of NK cells.^153^

Sampietro et al.^154^ assessed the immunomodulatory activity of delipidated extracts of propolis from the North of Argentina (mainly derived from *Salix humboldtiana* Willd., *Pinus* spp., and *Eucalyptus* spp.), and purified galangin and pinocembrin. Propolis was more effective as a chemotactic agent than the isolated compounds and stimulated higher neutrophil phagocytic activity, probably due to the synergistic effects among its components.^154^

Alanazi et al.^155^ evaluated in vitro the immunomodulatory activity of British poplar propolis ethanolic extract on murine bone marrow–derived macrophages co‐stimulated with LPS. The two samples used in the study had been collected in Essex and in the Midlands and were characterized by the presence of pinocembrin, pinobanksin, chrysin, galangin methyl ethers, and kaempferol (the last three being markedly higher in the sample from Essex). Propolis significantly reduced NO production and suppressed the secretion of IL‐1β and IL‐6, whereas the effect was less prominent on TNF‐α. The secretion of IL‐10 was also reduced, showing putative pro‐inflammatory effects suggestive of the enhancement of the immune response. Nonetheless, IL‐10 levels might be regulated by other cytokines, such as interferons, affected by propolis action. Slight differences among the two samples were attributed to the observation that flavones (chrysin) and flavonols (galangin and kaempferol) show a greater effect in inhibiting nitric oxide production by macrophages than flavanones (pinocembrin, pinobanksin). The metabolomic profiling, showing depletion of citrate, accumulated in M1 macrophages to sustain the inflammatory response, and an increase in itaconate, an anti‐inflammatory compound stimulating M2 polarization, supported the evidence of the inhibition of LPS‐induced NO production and suggested the hypothesis of differentiation toward an M2‐like phenotype.^155^

Oršolić et al.^156^ investigated the effect of a water‐soluble derivative of Croatian propolis and some isolated components (caffeic acid, CAPE, and quercetin) on the growth and metastatic potential of a transplantable mammary carcinoma of CBA mouse. The treatments were administered per os before the inoculation of tumoral cells. After 21 days the number of tumor nodules in the lungs was significantly lower, and propolis antimetastatic efficacy was higher than that of caffeic acid or CAPE. Quercetin was ineffective, but, if the treatment was protracted after tumoral cell inoculation, significant suppression of metastasis could be appreciated. In vitro, propolis extract did not influence HeLa cell growth, while caffeic acid and quercetin exerted a concentration‐dependent growth inhibition, revealing a direct antitumoral effect. The incubation of mammary carcinoma cells with test compounds led to the induction of apoptosis, maximal in the case of quercetin and CAPE, and necrosis. Since activated macrophages are thought to be a major component of the host defense against neoplastic growth, macrophages derived from treated mice were incubated with HeLa cells. The macrophages activated by propolis extract, caffeic acid, or CAPE inhibited DNA synthesis in HeLa cells. Moreover, treatment with propolis or CAPE increased macrophagic NO production.^156^ In the following work, the authors confirmed the inhibitory effects of propolis water‐soluble derivative, caffeic acid, and CAPE on mammary carcinoma metastasis formation, either preventively or curatively, showing that the antitumor activity is mostly related to the immunomodulatory properties of the compounds, their cytotoxicity to tumor cells, and their ability to induce apoptosis.^157^ In a mouse model of Ehrlich ascites tumor, orally administrated propolis water‐soluble derivative, caffeic acid, CAPE, and quercetin markedly reduced the volume of ascitic fluid and the total number of cells in the peritoneal cavity, prolonging the survival time of treated mice. The inhibition of tumor growth was due to the effect on the immune system since a dose‐related increase of cytotoxic T‐cell, NK, and B cell activity was observed in treated animals, as well as increased functional activity of macrophages in producing factors regulating the function of B‐, T‐, and NK cells.^158^ These studies on the antitumoral and antimetastatic effects, in which the immune system is unequivocally involved, fully demonstrate the potential of propolis as an immunomodulator able to stimulate immune functions, an effect which may find a useful application against viral and bacterial infections.

Mirzoeva et al.^159^ considered, instead, the activity of propolis ethanolic extract and its isolated components (caffeic acid, CAPE, quercetin, and naringenin) on eicosanoid production. In vitro, low concentrations (20–200 µg/ml) of propolis prevented LTB4, LTC4, and PGE2 secretion by murine peritoneal macrophages. Among the isolated compounds, CAPE strongly suppressed LTB4 and LTC4 synthesis in a concentration‐dependent manner, followed by caffeic acid and quercetin. Naringenin only slightly impaired LTC4 production. CAPE also had a concentration‐dependent inhibitory effect on PGE2 secretion. In vivo, in an acute peritoneal inflammation model in C57BL/6 mice, intraperitoneal administration of propolis extract decreased LTB4, LTC4, and PGE2 production by 95%, 90%, and 70%, respectively. CAPE inhibited LTB4 and LTC4 secretion, whereas caffeic acid affected only LTC4 synthesis. None of the isolated compounds was able to inhibit PGE2 production. The dietary supplementation of propolis during acute inflammation was also effective in reducing LTB4 and LTC4, but not PGE2, production.^159^ Rossi et al.^160^ investigated in vitro the effect of a commercial propolis ethanolic extract, as such and deprived of CAPE, and some isolated components on cyclooxygenase (COX‐1 and COX‐2) activity in J774.A1 murine macrophages. Propolis extract effectively inhibited PGE2 production by COX‐1 and COX‐2 in a concentration‐dependent manner. Among the isolated compounds, caffeic, ferulic, cinnamic, and chlorogenic acids, and pinocembrin did not affect the activity of COX isoforms, whereas CAPE and galangin showed inhibitory properties, the latter being about 10‐ to 20‐fold less potent. CAPE‐deprived propolis extract was about 10‐fold less potent than the extract as such in the inhibition of both COX‐1 and COX‐2, suggesting that CAPE and to a lesser extent galangin contribute to the overall activity of propolis.^160^

Finally, in their work Mounieb et al.^161^ examined in vivo the potential protective effect of propolis oral administration against liver injury in concanavalin A‐induced hepatitis in Wistar rats, a T‐cell‐dependent model that causes an immune‐mediated disease similar to the one induced by viral infections, in which oxidative stress, increased levels of TGF‐β, and fibrosis are the hallmarks. Induction with concanavalin A caused histopathological changes, reduction in serum albumin, and significant increase in serum levels of ALT, AST, and total bilirubin. Also, lipid peroxidation in liver tissue was found to be increased, while glutathione, and superoxide dismutase, and catalase activities were markedly downregulated. Serum levels of inflammatory cytokines (TNF‐α and IL‐6) and TGF‐β were increased. Propolis treatment was able to significantly attenuate all these deleterious effects, improving liver function.^161^

Altogether, these findings primarily suggest the anti‐inflammatory activity of propolis, frequently proven in activated, rather than resting, immune cells. The investigations on propolis isolated components recognized, when present, caffeic acid phenethyl ester (CAPE) as one of the main actors in propolis anti‐inflammatory actions. In addition, the enhancement of pathogen clearance and of the resistance to infections may be inferred.

### 4.5 Propolis as an immunological adjuvant

Several studies have examined the immunostimulatory potential role of propolis as an immunological adjuvant. Fan et al.^162^ investigated in vivo the immune‐enhancing efficacy of Chinese propolis flavonoids microemulsion on cyclophosphamide (CTX)‐induced immunosuppression and immune response in white Roman chickens. The treatment with propolis flavonoids microemulsion was able to overcome CTX‐induced immunosuppression and significantly increased the immune organ indexes, enhanced lymphocyte proliferation, and improved serum concentrations of IL‐2 and IL‐6. For the immune response experiment, the adjuvant effect of the formulation was evaluated on chickens intramuscularly immunized with recombinant Newcastle disease virus vaccine. The results demonstrated that propolis flavonoids microemulsion significantly promoted lymphocyte proliferation, enhanced antibody titers, and IgG/IgM concentrations.^162^ A liposomal preparation of Chinese propolis flavonoids could significantly enhance the phagocytic function of peritoneal macrophages harvested from ICR mice and stimulated IFN‐γ, IL‐1β, and IL‐6 production. In vivo, subcutaneous administration of the formulation with ovalbumin to mice could effectively activate the cellular and humoral immune response, increasing levels of IgG, IL‐4, and IFN‐γ in serum, and the proliferation rates of splenic lymphocytes.^163^ Sforcin et al.^164^ investigated the effect of Brazilian (collected in UNESP campus, Botucatu, São Paulo State) and Bulgarian propolis hydroalcoholic (70% ethanol) extracts, and two isolated compounds (caffeic acid and quercetin) on the antibody production in bovine serum albumin immunized rats. Both propolis types, independently from the collection season and geographical origin, stimulated antibody production in the same magnitude after 15 days of immunization, whereas caffeic acid and quercetin were ineffective at this purpose.^164^ Mojarab et al.^165^ investigated the adjuvant effects of aqueous and ethanolic extracts of propolis on a multi‐epitope recombinant vaccine against HIV‐1 in subcutaneously immunized BALB/c mice. Both extracts enhanced lymphocyte proliferation, IL‐4 and IFN‐γ production, and antibody responses with dominant IgG1 pattern, in a manner comparable to Freund's adjuvant or alum.^165^ In a series of studies, Fischer et al.^166^ evaluated in vivo the potentiality of green Brazilian propolis as an immunological adjuvant on the humoral immune response. The association of green Brazilian propolis ethanolic extract, containing artepillin C and cinnamic acid derivatives besides flavonoids, with an inactivated vaccine against bovine herpesvirus type 5 (BoHV‐5) resulted in a significant increase in the neutralizing antibody titers compared to control.^166^ The same extract was evaluated in BALB/c mice associated with inactivated suid herpesvirus type 1 (SuHV‐1) vaccine. The treatment with propolis extract alone did not induce significant levels of antibodies, an effect which could be obtained with the additional association of aluminum hydroxide. However, propolis was able to increase the cellular immune response, enhancing IFN‐γ mRNA expression.^167^ Finally, a phenolic compound‐rich fraction of green Brazilian propolis methanolic extract was further investigated to determine its effectiveness in the stimulation of cellular and humoral immune response when co‐administered with an inactivated vaccine against SuHV‐1. The treatment significantly increased neutralizing antibody titers against SuHV‐1, as well as the percentage of protected animals upon infection challenge, without requiring any other co‐adjuvant.^168^

Altogether, these results demonstrate that propolis extracts of different types, among which the most studied are of Chinese and Brazilian origin, possess intriguing immunostimulatory properties that could be effectively exploited to enhance humoral immunity. Therefore, the utility of propolis as an alternative and more efficient immunological adjuvant should be first confirmed in preclinical studies, propaedeutic to future clinical trials aimed at assessing the relevance of this mechanism.

## 5 PROPOLIS APPLICATIONS IN RESPIRATORY DISEASES

An abnormal inflammation of the respiratory tract can be a life‐threatening condition. Several pathologies may cause breathing difficulties, but more commonly the respiratory tract is highly reactive toward airborne toxic pollutants, cigarette smoke, irritant agents, allergens, and pathogens including viruses. A persistent inflammatory response might be due to an impairment in macrophage clearance from the inflamed site, chronic infection, oxidative stress, or local hypoxia. All these stimuli can lead to tissue remodeling of the airway walls, and definitely impair gas exchange at the level of the blood–air barrier.^169^

Inflammatory responses are triggered by a complex interaction between neutrophils and epithelial cells, stimulating the recruitment of immune cells to the injured site. TNF‐α and IL‐8 are involved in the first phase of inflammation, increasing the expression of adhesion molecules on the endothelial cells of lung capillaries and attracting neutrophils, respectively.^170^ Other inflammatory mediators generally expressed in respiratory tract inflammatory processes are MMP‐9, the adhesion molecules ICAM‐1 and VCAM‐1, COX‐2, and cytosolic phospholipase A2.^171^ In particular, metalloproteinases cause the degradation of the extracellular matrix, possibly contributing to the onset of emphysema,^172^ and play a significative role in the process, regulated by TGF‐β, of airway tissue remodeling during asthma.^173^

In the following paragraphs, the studies which demonstrate the ability of propolis, or its isolated components, to modulate the immune and inflammatory responses in the respiratory tract are presented.

Besides the clinical studies on propolis antiviral properties (Crişan et al.,^60^ Cohen et al.,^61^ Szmeja et al.,^62^ Esposito et al.,^63^ Di Pierro et al.,^65^ and Silveira et al.^77^) described in the previous sections, few other works focused on the potential efficacy of propolis for the treatment of human respiratory diseases. Khayyal et al.^174^ conducted a clinical study to assess the beneficial effects of a propolis‐based food supplement as an adjuvant in adult patients with mild to moderate asthma treated with oral theophylline. The formulation contained an aqueous extract of propolis collected in Denmark, China, Uruguay and Brazil, and standardized to contain not less than 0.05% of aromatic acids (mainly caffeic, ferulic, isoferulic, cinnamic, and 3,4‐dimethoxy‐cinnamic acids), in addition to trace amounts of various flavonoids. After 2 months of treatment, patients receiving propolis showed a marked reduction in the incidence and severity of nocturnal attacks (from an average of 2.5 per week to only one) and an improvement of ventilatory functions. The serum levels of the pro‐inflammatory cytokines TNF‐α, ICAM‐1, IL‐6, and IL‐8 dropped by 52%, 65%, 44%, and 30%, respectively, whereas IL‐10 increased by 3‐fold. PGE2, PGF2α, and LTD4 were significantly decreased to 36, 39, and 28%, respectively, of initial values.^174^ This effect is consistent with the evidence concerning immunomodulatory effects presented in the previous section.

Although not properly being a respiratory disease, the consequences of the exposure to air pollutants on the human organism partially involve the respiratory tract. Sojka et al.^175^ conducted a study on 440 children, natives of a heavily polluted region around Legnica (Poland), during a stay in the health‐resort region of Rabka. Acute‐phase proteins levels were found to differ significantly with respect to ferritin and transferrin serum levels in comparison with local children. Treatment with propolis significantly modified the climatic effects on erythrocyte sedimentation rate, electrophoretic fractions of serum proteins, and some of the acute phase proteins involved in iron metabolism.^175^

A few studies have investigated the effects of various propolis samples against bacterial pathogens implicated in lung infections. The oral administration of propolis significantly decreased the levels of malondialdehyde, an oxidative stress marker, and restored those of superoxide dismutase, strengthening the endogenous antioxidant system in a model of endotoxin‐induced acute lung inflammation in rats.^176^ De Marco et al.^177^ examined the antibacterial activity of Italian propolis hydroalcoholic (85% ethanol) extract, characterized by the presence of chrysin, galangin, pinocembrin, and CAPE, against *Pseudomonas aeruginosa*, an opportunistic pathogen with the ability to form biofilms majorly associated with chronic lung infection. The minimum inhibitory concentration (MIC) of the extract was determined at 125 µg/ml, and the effect was bacteriostatic. In addition, propolis was able to reduce biofilm formation and diminished the number of alive *P. aeruginosa* cells in the biofilm, suggesting that the reduction of biofilm mass was due to a diminished number of sessile bacteria. In particular, propolis partially inhibited the swimming activity of *P. aeruginosa* and the formation of a stable adhesion, while no effect on swarming and twitching activity could be observed.^177^ The protective effect of the oral administration of a Saudi red propolis aqueous extract against lung damage was assessed in rats that were intraperitoneally injected with methicillin‐resistant *Staphylococcus aureus*. Propolis ameliorated the oxidative stress biomarker malondialdehyde, as well as the antioxidant markers glutathione peroxidase and superoxide dismutase. Moreover, propolis extract modulated the alterations in TNF‐α and VEGF serum levels and ameliorated oxidative DNA damage and the apoptosis biomarker caspase‐3 in the lungs. The biochemical results were supported by the histopathological observation of lung tissue.^178^ Sayed et al.^179^ evaluated the oral and intraperitoneal administration of Egyptian propolis hydroalcoholic (70% ethanol) and aqueous extracts in Sprague‐Dawley rats for 60 days before intraperitoneal injection of *S. aureus*. In control infected rats, the lung was the most affected organ, showing several focal pus nodules on the visceral surface of pleura and suppurative bronchopneumonia. The bronchial epithelium was degenerated and destructed. In the treated group, 8.6% of the rats had morbidity manifestations; however, areas with signs of pneumonia were smaller with lower leukocytic infiltration, and bronchi were unaffected. The remaining rats (91.4%) were asymptomatic, and their lungs were microscopically normal without any lesion. Bacterial re‐isolation could also not be obtained.^179^ On the other hand, when the effect of the oral administration of propolis was evaluated in a rat model of *Pneumocystis carinii* infection, the treatment was found to be completely ineffective in reducing the number of cysts in the lungs of infected animals.^180^ However, in the context of bacterial infections propolis has generally proven to exert both direct antibacterial activity, and anti‐inflammatory and immunomodulatory effects, which seem particularly relevant in conditions that constitute a challenge for the immune system, such as disseminated infections and endotoxemia, and are characterized by systemic detrimental outcomes.

Barroso et al.^181^ tested the hypothesis that the oral supplementation of Brazilian propolis ethanolic extract would repair lung damage in emphysema caused by cigarette smoke exposure. C57BL/6 mice were exposed to cigarette smoke for 60 days, then treated with propolis for additional 60 days. Histological analysis revealed the ability of propolis to reverse the septum and alveolar destruction, significantly improving lung histoarchitecture. Additionally, the extract increased MMP‐2 and decreased MMP‐12 expression, enhancing the process of tissue repair and leukocyte recruitment. In particular, propolis promoted macrophage alternative activation, increasing IL‐10 levels and favoring an anti‐inflammatory microenvironment, paralleled by the downregulation of IGF1 expression in a Nrf2‐independent manner.^181^ The efficacy of the oral administration of green Brazilian propolis ethanolic extract against acute lung inﬂammation induced by cigarette smoke was assessed by Lopes *et al*. Propolis treatment reduced the number of alveolar macrophages and neutrophils, and restored NO, malondialdehyde and myeloperoxidase levels, and antioxidant enzyme activity in lung homogenates. Additionally, TNF‐α expression was reduced.^182^

Pulmonary fibrosis, a condition caused by an alteration in the levels of fibrogenic mediators, is the most common outcome of different lung disorders known as interstitial lung diseases. Bilgin et al.^183^ investigated the protective effects of Turkish propolis oral administration in a model of bleomycin‐induced lung fibrosis in Wistar rats. Propolis lowered oxidative stress markers (malondialdehyde and myeloperoxidase levels) and increased catalase activity. Although mean fibrosis scores were not significantly different, the ultrastructural investigation revealed that propolis diminished lung fibrosis more effectively than prednisolone.^183^ Ismail and Farag^184^ investigated the protective role of propolis oral administration against bleomycin‐induced pulmonary fibrosis in male albino rats. Bleomycin‐treated animals showed significantly decreased glutathione and increased malondialdehyde levels, complete loss of normal lung architecture (septal thickening, congestion of blood vessels, inflammatory infiltration, and increased number of fibroblasts), with a significant increase in collagen fibers and α‐SMA (smooth muscle actin), iNOS, and cytochrome c immunoreactivity. Propolis‐treated animals revealed the restoration of glutathione levels and a significant reduction of malondialdehyde, nearly normal lung architecture, and amelioration of all the other alterations.^184^ Kao et al.^185^ assessed the inhibitory effect of Brazilian propolis hydroalcoholic (70% ethanol) extract on TGF‐β1‐induced epithelial–mesenchymal transition in human alveolar epithelial cells, a condition suggested to possibly contribute to airway remodeling in fibrotic lung diseases and severe asthma. A549 cells pretreated with propolis and then treated with TGF‐β1 retained epithelial cell morphology, decreased N‐cadherin and ROS production, and showed reduced motility. In particular, propolis prevented the TGF‐β1‐induced downregulation of PPARγ protein, suggesting that the anti‐fibrotic effect of propolis extracts may, at least in part, be mediated by PPARγ activation. CAPE and pinocembrin exerted no inhibitory effect on TGF‐β1‐induced morphological changes in A549 cells, but had only partial effects on epithelial‐mesenchymal transition, suggesting the involvement of other unidentified components of propolis which may act as PPARγ ligands.^185^ Altogether, these findings suggest the beneficial role of propolis in pathological conditions that involve the disruption of lung tissue equilibrium with loss of histoarchitecture and onset of fibrosis, such as the consequences of viral or bacterial respiratory infections which induce an over‐reaction of the immune system at pulmonary level.

The study by El‐Aidy et al.^186^ aimed at examining the modulatory effects of the intraperitoneal administration of Egyptian propolis ethanolic and aqueous extracts on lung inflammation in a model of conalbumin‐induced asthma in CD1 mice. The percentage of circulating and lung‐infiltrated eosinophils and basophils were significantly decreased in mice treated with propolis. In addition, histopathological examination of lungs revealed signiﬁcant decreases in inﬂammatory scores, suggesting considerable ameliorative effects against asthma.^186^ Sy et al.^187^ investigated the immunoregulatory and anti‐inflammatory activities of Taiwanese propolis aqueous extracts in a model of ovalbumin‐induced asthma in BALB/c mice. Propolis oral administration was effective in suppressing the serum levels of ovalbumin‐specific IgE and IgG1, and airway hyperresponsiveness in sensitized mice. Although no significant differences were found in the concentration of eotaxin or eosinophil number in bronchoalveolar lavage fluid, the higher doses of propolis could decrease IL‐5 levels. In addition, cytokine (IFN‐γ, IL‐6, and IL‐10) secretion in ovalbumin‐stimulated splenocytes from propolis‐treated mice was significantly lower compared to the controls.^187^ A standardized green propolis extract (EPP‐AF®) was studied in C57BL/6 mice challenged with allergens. The extract was characterized by the presence of high quantities of artepillin C, 4,5‐dicaffeoylquinic acid, drupanin, 3,5‐dicaffeoylquinic acid, and *p*‐coumaric acid. Propolis treatment decreased the total cell number and IL‐5 levels in bronchoalveolar lavage fluid, and IL‐13 levels in lung tissue, confirming the attenuation of T_h_2 inflammation. M2 macrophage number was also reduced in the lungs of treated mice. Moreover, propolis enhanced the frequency of polymorphonuclear myeloid‐derived suppressor, which can induce T_reg_ differentiation. Accordingly, an increase in frequency and total number of CD4^+^ Foxp3^+^ T_reg_ cells could be observed. Interestingly, this effect could be elicited directly by propolis on isolated T‐cells in vitro.^188^ The preclinical evidence of propolis effectiveness in models of asthma and allergy corroborates the results obtained in the above‐mentioned clinical investigations.

Hu et al.^189^ explored the anti‐inﬂammatory effects of Chinese poplar propolis hydroalcoholic (80% ethanol) and aqueous extracts in a model of carrageenan‐induced pleurisy and LPS‐induced acute lung damage in rats. Both extracts exerted significant effects on pleural effusion, leukocyte counts, and NO and PGE2 levels in carrageenan‐induced pleurisy. Propolis inhibited lung edema in the acute damage model, but the results were not significant. In addition, the hydroalcoholic extract showed a good ability to counteract the increase in neutrophils and the decrease in lymphocytes in lungs.^189^ In a model of lung injury induced by oleic acid and LPS injection in Wistar rats, propolis ethanolic and aqueous extracts antagonized lung edema, decreased inflammation, and inhibited the expression and activation of NF‐κB p65 subunit.^190^ Borrelli et al.^191^ investigated the role of the intraperitoneal administration of an Italian propolis ethanolic extract, as such and deprived of CAPE, galangin, and CAPE in a model of carrageenan‐induced pleurisy in rats. The treatment with propolis as such and CAPE produced a significant dose‐dependent reduction of exudate volume and an inhibition of leukocyte migration into the pleural cavity. On the other hand, galangin and the extract deprived of CAPE were completely ineffective.^191^ These findings call directly for propolis anti‐inflammatory properties, and once again demonstrate that, whenever present, CAPE seems to be a major determinant of such effects.

Mechanical trauma of the nasal mucosa, especially after chronic rhinosinusitis, may cause excessive encrustation and eventually synechia, a condition characterized by adherences between turbinates, or turbinates and lateral wall/septum. Propolis oral administration, aimed at healing the injured nasal mucosa in Wistar rats, reduced the degree of inflammation and prevented ciliated and goblet cell loss, preserving the histological integrity of the tissue.^192^

Sobočanec et al.^193^ evaluated the preventive effect of the dietary supplementation of raw Croatian propolis, rich in chrysin, pinocembrin, and galangin, in CBA/Hr mice subjected to hyperoxia. Despite possessing a relatively low antioxidant capacity in vitro, propolis proved to be a strong antioxidant in vivo. Since lungs of adult animals are vulnerable to hyperoxia due to their incapacity to rapidly augment antioxidant defenses, oxidative stress significantly increased lipid peroxidation by‐product levels. Propolis abrogated such an increment, thanks to the enhancement of manganese‐dependent superoxide dismutase and catalase activity. Altogether these results suggest the possibility to exploit propolis strong antioxidant and scavenging abilities in case of prolonged oxygen therapy.^193^

Finally, Rossi et al.^194^ investigated the role of an Italian propolis ethanolic extract characterized by high CAPE content (10.44%), as such and deprived of CAPE, and some isolated compounds (CAPE, galangin, pinocembrin, caffeic, chlorogenic, ferulic, and cinnamic acids) in the inhibition of lung cyclooxygenase activity. Propolis as such (0.3–300 µg/ml), CAPE (0.1–100 µM), and galangin (1–100 µM) significantly inhibited prostanoid generation by rat lung cyclooxygenases in a concentration‐dependent manner, with and without LPS induction. The extract deprived of CAPE was approximately 10‐fold less potent. These results suggest that both CAPE and galangin contribute to the overall inhibitory activity of propolis; however, CAPE is the principal contributor, since the inhibition curves of propolis as such and CAPE were almost superimposable.^194^

Although only a few clinical studies have been conducted to assess propolis efficacy against respiratory diseases, namely viral upper respiratory tract infections, asthma, and pollution‐related inflammatory states, a plethora of preclinical, mainly in vivo, evidence has demonstrated the beneficial effects of this natural product in such pathological context. Propolis has proven its efficacy against bacterial infections, showing direct antibacterial activities, but also more intriguing immunomodulatory and anti‐inflammatory effects that allow the preservation of lung histoarchitecture and function. Similar effects were demonstrated also in different other models of respiratory diseases, such as smoke‐related emphysema, fibrosis, asthma, as well as pleurisy and generic lung injury, outlining the action profile of a wide spectrum therapeutic agent.

### 5.1 Propolis and COVID‐19

The severity of COVID‐19, the disease caused by the recent novel coronavirus (SARS‐CoV‐2) outbreak and characterized by high plasma levels of inflammatory mediators (e.g., IL‐1β, IL‐8, IFN‐γ, MCP1, and TNF‐α) ^195^ and symptoms of acute respiratory distress, has brought to the fore again the significant role of inflammation and the so‐called “cytokine storm” in the clinical outcomes of respiratory diseases.^55^, ^56^ In a retrospective study on patients with severe COVID‐19, serum levels of IL‐6, IL‐8, and TNF‐α were significantly higher compared to patients with mild disease.^196^ Such an increase in levels of pro‐inflammatory cytokines and chemokines is strongly associated with organ dysfunction more than the actual viral titers and leads to the development of respiratory impairment and pulmonary failure.^197^, ^198^

Based on that, the last months have seen a renewed interest in the use of propolis in the COVID‐19 pandemic, also thanks to the emergence of novel potential targets in SARS‐CoV‐2 infection mechanism.^199^ SARS‐CoV‐2 entry into the host cell is mediated by the interaction between angiotensin‐converting enzyme 2 (ACE2) and viral spike (S) glycoproteins, primed by the host serine protease TMPRSS2.^200^ Following viral entry, the pathogenesis is characterized by the upregulation of PAK1, a serine‐threonine protein kinase with a nodal signaling role, involved in the suppression of the adaptive immune response, lung inflammatory processes, and fibrosis when abnormally activated.^201^

Many naturally occurring compounds, including propolis components such as quercetin, myricetin, and caffeic acid, have been selected in in vitro screenings and exploiting virtual docking models as promising antiviral agents able to bind and inhibit SARS‐CoV‐2 key proteins. Interestingly, the majority of drug candidates are polar compounds, structurally belonging to the class of polyphenols.^202^ Among the potential targets, SARS‐CoV‐2 M^pro^ is a 3‐chymotripsin‐like cysteine enzyme necessary for the processing of viral polyproteins. Hashem used an in silico molecular modeling approach to evaluate the activity of different propolis components, concluding that caffeic acid, CAPE, galangin, and chrysin possess a strong binding affinity and may inhibit the main protease of SARS‐CoV‐2.^203^ In a similar work by Kumar et al.,^204^ once again CAPE was predicted to possibly interact with SARS‐CoV‐2 M^pro^. In an in silico screening of the possible activity of Egyptian propolis against SARS‐CoV‐2 M^pro^, RNA‐dependent RNA‐polymerase (RdRp), and spike protein subunit 1 (S1), Elwakil et al.^205^ showed that octatriacontyl pentafluoropropionate, a component identified in propolis from Menoufia by GC/MS analysis, displays excellent orientation and binding capacity in the active site of the target macromolecules, emerging as a promising broad‐spectrum antiviral agent. Harisna et al.^206^ performed a molecular docking simulation to assess the ability of 22 compounds found in Indonesian propolis to inhibit SARS‐CoV‐2 M^pro^ and spike protein subunit 2 (S2). Methylophiopogonone A, 3′‐methoxydaidzin, and genistin were shown to be candidate inhibitors of M^pro^. Although only 3′‐methoxydaidzin displays a binding affinity comparable to the control nelfinavir, all these compounds presented a key hydrogen bond at residue GLU A:166, similarly to nelfinavir. On the other hand, neoblavaisoflavone, methylophiopogonone A, 3′‐methoxydaidzin, and genistin all presented a predicted binding affinity against S2 far below the control pravastatin.^206^ Finally, Shaldam et al.^207^ discovered by molecular docking analysis the significative binding affinity of 14 propolis phenolics and terpenes against M^pro^ and RdRp. Among these compounds, ellagic acid, hesperetin, and kaempferol were the most promising RdRp inhibitors, while artepillin C, ellagic acid, hesperetin, kaempferol, and quercetin had the strongest interactions with M^pro^.^207^

Besides viral targets, propolis possesses the potentiality to interact also with the host proteins of the cell surface involved in the infection mechanism. Recent evidence showed that a TMPRSS2 inhibitor approved for clinical use could block viral entry, bringing to light an innovative treatment option.^208^ Kaempferol, in fact, had been previously shown able to decrease the mRNA expression of TMPRSS2.^209^ The inhibition of ACE2 may also be a relevant target against SARS‐CoV‐2 infection and has been suggested as a therapeutic alternative. Despite the possible drawbacks of the use of ACE inhibitors in patient with COVID‐19, a recent observational study dispelled the doubts, and this class of drugs remains an important tool against potential cardiovascular complications.^210^ Güler et al.^211^ determined the composition of Anatolian propolis hydroalcoholic (70% ethanol) extracts in terms of phenolic acids and flavonoids and screened them as ligands for ACE2 receptors in molecular docking analysis. The results showed that rutin had the best inhibitory potential, followed by myricetin, CAPE, hesperetin, and pinocembrin. In particular, rutin could interact with zinc finger residues of the active sites of ACE2.^211^ In addition, Osés et al.^212^ evaluated in vitro several hydroalcoholic extracts (70% ethanol) of propolis from different origins (North‐East Europe, South‐West Europe, and South America) for their ACE inhibitory activity, which resulted higher than 95% for all the samples, except for one with low flavanol content. The higher values of ACE inhibition were found in samples with higher amounts of catechin and *p*‐coumaric acid.^212^

As previously stated, the pathogenesis of COVID‐19 seems dependent on PAK1 abnormal activation, responsible for the suppression of the host immune system and lung fibrosis.^201^ In addition PAK1 is a critical mediator of the “cytokine storm” that frequently is a cause of mortality in hospitalized patients.^213^ The downregulation of PAK1 activity could restore the immune response, boosting the antibody production against SARS‐CoV‐2. At a molecular level, PAK1 is directly activated by Rac1, a G protein belonging to the Rho family of GTPases. Xu et al.^214^ demonstrated that caffeic acid could reduce Rac1 protein and activity level, thus inhibiting downstream PAK1 activation. CAPE, green Brazilian propolis extract, and artepillin C can also selectively inhibit PAK1.^215^ Finally, the inhibitory effect of Pacific propolis from *Macaranga tanarius* (L.) Müll. Arg., in particular the Okinawan type, on PAK‐1 has recently been reviewed by Shahinozzaman and colleagues.^216^

In recent times, some clinical evidence has been added to in vitro and computational findings. Fiorini et al.^217^ examined the case of a 52‐year‐old female patient suffering from COVID‐19 who self‐administered a nonalcoholic preparation of green Brazilian propolis extract EPP‐AF®, at a dose of 45 drops three times a day for 14 days. After 12 days of treatment, the patient had recovered and the nasopharyngeal swab gave a negative RT‐PCR test result.^217^ The relevance of a single case report is debatable, but in light of the previously described clinical trial conducted by Silveira et al.^77^ in the same period (June–August 2020), this first report of a possible clinical efficacy of propolis against COVID‐19 contributes to supporting the therapeutic potential of green Brazilian propolis, and by extension propolis in general, against SARS‐CoV‐2 infection.

## 6 CONCLUSION

Propolis is a collective term used to define a multifaceted bee product outwardly uniform in physical appearance, gross composition, and purpose, but dramatically diverse in its chemical components. From an ecological point of view, foraging for resins by honeybees is an energetically demanding activity that provides no clear individual reward. However, it should not be forgotten that several plant species secrete resins and other exudates with antimicrobial properties in response to tissue damage, to protect buds, vegetal apices, and young leaves. Thus, honeybees have probably evolved the ability to collect antimicrobial compounds from the surrounding environment as a means of reducing the deleterious effects of parasites and pathogens by enhancing a colony‐level social immunity, rather than investing only in individual immune defenses.^218^ Starting from this premise, it seems clear that despite an extreme chemical variability, which depends on the botanical sources around the beehive and poses a serious issue about the product standardization, propolis possesses general pharmacological properties in terms of antimicrobial activity, which represent one of the main reasons for its clinical use against human diseases. Moreover, resins and exudates gathered by honeybees contain also a variety of plant secondary metabolites, mainly of phenolic nature, renowned for their anti‐inflammatory and immunomodulatory activities. Therefore, propolis is a “super‐blend” of biologically active compounds evolutionarily sorted by honeybees, which pharmacological research can and should exploit to its advantage. Nevertheless, besides propolis multifariousness, there is a common thread among the chemical compositions of different samples showing similar biological activities: high concentrations of flavonoids and/or phenolic acids (mainly cinnamic and hydroxycinnamic acids, and their derivatives). As reported in this review, the most studied, and probably most promising, propolis types are the poplar and the green Brazilian ones, which are indeed characterized by flavonoids and by hydroxycinnamic acids and their prenylated derivatives, respectively.

All the collected in vitro, ex vivo, and in vivo evidence supports the hypothesis that propolis (both hydroalcoholic/ethanolic or aqueous extracts, although with slightly different mechanisms due to the different composition) may exert a direct or indirect broad‐spectrum antiviral activity against several different viral families. On the one hand, in fact, propolis seems able to interfere directly, most often nonspecifically, with viral particles or steps in the viral replication cycle. On the other, it stimulates and strengthens the host innate and adaptive resistance against viral infections. Propolis and its isolated components have been shown to exert immunosuppressive activities, possibly related to the anti‐inflammatory properties, on different T lymphocytes subsets, but paradoxically activate macrophagic and NK cell functions. And perhaps this is the most fascinating and promising effect. As we now know, the most devastating consequences of viral diseases often arise from a dysregulated response of the immune system and the consequent imbalance in cytokine and chemokine production.^55^, ^56^ In this context, propolis may demonstrate a dual nature as immunostimulant, to prevent the infection, and immunomodulator, to dampen the inflammatory state and counter the immune dysfunction after the onset of the disease.

As to clinical studies, almost all trials assessing propolis antiviral efficacy focused on poplar type propolis collected in temperate regions and, despite the variety of viral targets in the preclinical studies, considered a very narrow range of viral diseases. The overall highest‐quality evidence is about propolis use in the treatment of herpes labialis. Most of the studies are double‐blind, controlled, randomized clinical trials that demonstrate the superiority of propolis‐based ointments, creams, and lip‐balms in shortening the healing time and improving the clinical course. In addition, similar results were obtained with two different propolis extracts (ACF® and GH 2002), reinforcing the value of propolis as an effective natural product in spite of its individual chemical composition. For this reason, formulations containing flavonoid‐rich propolis extract might be strongly recommended for the clinical management of herpes labialis. In the case of genital herpes and shingles, clinical studies provide moderate‐ to low‐quality evidence. Nevertheless, considering that all these viral species belong to the same *Herpesviridae* family, the use of propolis may be recommended, at least as an add‐on therapy to conventional pharmacological treatment. The studies regarding other viral families are often scarce and provide very low‐quality evidence. Nonetheless, whenever data were still insufficient to substantiate an activity against certain viral families, there are substantial premises that suggest a potential antiviral efficacy. Therefore, further investigations are still needed before making a judgment of propolis efficacy and should be warmly encouraged. Conversely, although solitary, a well‐designed clinical trial demonstrated the disease‐modifying role of a blend of poplar and *Baccharis* spp. propolis in dengue hemorrhagic fever. In this case, the most likely mechanism of action is the anti‐inflammatory and immunomodulatory one, supported by the evidence of a reduction in pro‐inflammatory cytokine levels.

As previously stated, propolis is a complex product with a broad spectrum of activity that goes far beyond the simple sum of its isolated components. At the current state of knowledge, due to the animal origin and the extreme variability, with all the associated standardization problems, propolis is therefore unlikely to reach the status of medicinal product recognized by regulatory agencies in the near future. Nevertheless, propolis is already available as a dietary supplement in many countries, and a plethora of preclinical studies and some clinical studies^60^, ^62^, ^65^, ^76^, ^174^ considered in this review, involving the assessment of propolis systemic effects, have successfully explored the possibilities of an oral administration. Starting from these preliminary remarks, possible use for the prevention of human respiratory diseases may be inferred. Furthermore, with respect to the recent outbreaks of pandemic respiratory viral infections, propolis supplementation could be put to good use as an add‐on therapy to help control viral replication and infectivity, and, above all, counteract the deleterious inflammatory drawbacks on the respiratory tract. Propolis clinical efficacy against certain viral diseases has yet been demonstrated,^60^, ^62^, ^65^ and is supported by the preclinical evidence of a general broad‐spectrum antiviral activity. Moreover, propolis possesses additional antibacterial properties,^176^, ^177^, ^178^, ^179^ useful against opportunistic superinfections. The modulatory effects on the balance between pro‐ and anti‐inflammatory cytokines, together with those exerted on different immune cell subtypes, corroborate the positive effects on lung histoarchitecture in case of emphysema and fibrosis,^181^, ^182^, ^183^, ^185^, ^219^, ^220^, ^221^ or acute respiratory distress.^222^ Finally, it should not be forgotten the possibility to exploit propolis strong antioxidant and radical scavenging abilities in case of prolonged oxygen therapy.^193^

For what specifically concerns COVID‐19, propolis immunomodulatory activity may be exploited in the initial phases of SARS‐CoV‐2 infection, characterized by the dysregulation of the immune response which facilitates viral replication, to reduce immunosuppression. Conversely, in later stages of the disease, propolis might counteract the onset of an exaggerated inflammatory response, hallmarked by a “cytokine storm” which causes multiorgan failure and makes it necessary to resort to intensive care and the use of ventilators.^223^ Moreover, propolis may directly interfere with viral infectivity and replication, hindering the interaction between the virus and the host cell by targeting key proteins on both sides. Nevertheless, despite the encouraging results obtained in the first clinical trial conducted against COVID‐19 with green Brazilian propolis,^77^ stronger preclinical evidence is still required to corroborate such a therapeutic approach, not to mention that the effective bioavailability of propolis components at target organs in vivo is yet to be proven, being also possible that metabolites are responsible for the biological effects.

Until now, only a few clinical studies have evaluated the putative efficacy of propolis oral administration in the case of respiratory diseases, but none of them was conducted as a rigorous double‐blind, controlled, randomized trial. Moreover, study backgrounds and propolis extracts differed significantly, thus providing low‐quality evidence of efficacy which unfortunately prevents from formulating even a weak recommendation. Nonetheless, preclinical studies support the all‐round potential of propolis in respiratory diseases and, given the current emergency caused by the COVID‐19 pandemic and the limited therapeutic options, propolis should be suggested as a reasonably safe and hopefully effective therapeutic agent.

For all these reasons, it would be advisable that rigorous randomized clinical trials be conducted to ascertain propolis efficacy against respiratory tract infections and resultant diseases, opening up new perspectives for the rational use of propolis as an effective dietary supplement, or even a fully‐fledged therapeutic agent in the future.

## Data Availability

The data used to support the findings of this study are available from the corresponding author upon request.
